# Identification of potential inhibitors based on compound proposal contest: Tyrosine-protein kinase Yes as a target

**DOI:** 10.1038/srep17209

**Published:** 2015-11-26

**Authors:** Shuntaro Chiba, Kazuyoshi Ikeda, Takashi Ishida, M. Michael Gromiha, Y-h. Taguchi, Mitsuo Iwadate, Hideaki Umeyama, Kun-Yi Hsin, Hiroaki Kitano, Kazuki Yamamoto, Nobuyoshi Sugaya, Koya Kato, Tatsuya Okuno, George Chikenji, Masahiro Mochizuki, Nobuaki Yasuo, Ryunosuke Yoshino, Keisuke Yanagisawa, Tomohiro Ban, Reiji Teramoto, Chandrasekaran Ramakrishnan, A. Mary Thangakani, D. Velmurugan, Philip Prathipati, Junichi Ito, Yuko Tsuchiya, Kenji Mizuguchi, Teruki Honma, Takatsugu Hirokawa, Yutaka Akiyama, Masakazu Sekijima

**Affiliations:** 1 Education Academy of Computational Life Sciences (ACLS), Tokyo Institute of Technology, 4259 Nagatsutacho, Midori-ku, Yokohama 226-8501 Japan; 2 Level Five Co. Ltd., Shiodome Shibarikyu Bldg., 1-2-3 Kaigan, Minato-ku, Tokyo 105-0022, Japan; 3 Department of Computer Science, Tokyo Institute of Technology, 2-12-1, Ookayama, Meguro-ku, Tokyo 152-8550 Japan; 4 Department of Biotechnology, Bhupat and Jyoti Mehta School of Biosciences, Indian Institute of Technology Madras, Chennai 600 036, Tamilnadu, India; 5 Department of Physics, Chuo University, 1-13-27 Kasuga, Bunkyo-ku, Tokyo 112-8551, Japan; 6 Department of Biological Sciences, Chuo University, 1-13-27 Kasuga, Bunkyo-ku, Tokyo 112-8551, Japan; 7 Okinawa Institute of Science and Technology Graduate University, 1919-1 Tancha, Onna-son, Kunigami, Okinawa 904-0495 Japan; 8 The Systems Biology Research Institute, Falcon Building 5F, 5-6-9 Shirokanedai, Minato-ku, Tokyo 108-0071 Japan; 9 Center for Integrative Medical Sciences, RIKEN, 1-7-22 Suehiro-cho, Tsurumi-ku, Yokohama City, Kanagawa, 230-0045, Japan; 10 Research Center for Advanced Science and Technology, The University of Tokyo, 4-6-1 Komaba, Meguro-ku, Tokyo 153-8904 Japan; 11 PharmaDesign Inc., 2-19-8, Hatchobori, Chuo-ku, Tokyo 104-0032 Japan; 12 Department of Computational Science and Engineering, Nagoya University, Furocho, Chikusa, Nagoya 464-8603, Japan; 13 Division of Neurogenetics, Nagoya University Graduate School of Medicine, 65 Tsurumai, Showa-ku, Nagoya 466-8550, Japan; 14 Information and Mathematical Science and Bioinformatics Co., Ltd., Level 6 OWL TOWER, 4-21-1 Higashi-Ikebukuro, Toshima-ku, Tokyo 170-0013 Japan; 15 Global Scientific Information and Computing Center, Tokyo Institute of Technology 2-12-1, Ookayama, Meguro-ku, Tokyo 152-8550 Japan; 16 Department of Biotechnology, The University of Tokyo, 1-1-1 Yayoi, Nunkyo-ku, Tokyo, 113-8657; 17 Forerunner Pharma Research, Co., Ltd., Yokohama Bio Industry Center, 1-6 Shuehiro-cho, Tsurumi-ku, Yokohama 230-0045 Japan; 18 Centre of Advanced Study in Crystallography and Biophysics and Bioinformatics Infrastructure Facility (DBT Funded), University of Madras, Chennai 600025, Tamilnadu, India; 19 National Institutes of Biomedical Innovation, Health and Nutrition, 7-6-8 Saito-Asagi, Ibaraki, Osaka 567-0085 Japan; 20 Center for Life Science Technologies, RIKEN, 6-7-3 Minatojima-minamimachi, Chuo-ku, Kobe-shi, Hyogo 650-0047 Japan; 21 Molecular Profiling Research Center for Drug Discovery, National Institute of Advanced Industrial Science and Technology, 2-4-7 Aomi, Koto-ku, Tokyo, 135-0064, Japan; 22 Initiative for Parallel Bioinformatics, Level 14 Hibiya Central Building, 1-2-9 Nishi-Shimbashi Minato-Ku, Tokyo 105-0003 Japan

## Abstract

A search of broader range of chemical space is important for drug discovery. Different methods of computer-aided drug discovery (CADD) are known to propose compounds in different chemical spaces as hit molecules for the same target protein. This study aimed at using multiple CADD methods through open innovation to achieve a level of hit molecule diversity that is not achievable with any particular single method. We held a compound proposal contest, in which multiple research groups participated and predicted inhibitors of tyrosine-protein kinase Yes. This showed whether collective knowledge based on individual approaches helped to obtain hit compounds from a broad range of chemical space and whether the contest-based approach was effective.

Novel drug discovery is generally considered to be a time-consuming and expensive process. A typical drug discovery process takes 12–14 years and costs approximately one billion dollars^1^,^2^. Various approaches have been developed to explore promising drug candidates while reducing the financial and time burdens imposed in acquiring new molecular entities. Techniques such as combinatorial chemistry and high-throughput screening have been used in traditional drug development^3^,^4^. Since the 1960s, the available scientific knowledge has been used to guide drug discovery, and computer-aided drug discovery (CADD) is currently a highly efficient technique in achieving these objectives. In the post-genomic era, CADD can be combined with data from large-scale genomic amino acid sequences, three-dimensional (3D) protein structures, and small chemical compounds and can be used in various drug discovery steps, from target protein identification and hit compound discovery to the prediction of absorption, distribution, metabolism, excretion, and toxicity (ADMET) profiles^5^,^6^,^7^. The use of CADD is expected to cut drug development costs by 50%^8^. CADD approaches are divided into two major categories: protein structure-based (SB) and ligand-based (LB) methods. The SB approach is generally chosen when high-resolution structural data such as X-ray structures are available for the target protein. The LB approach is used to predict ligand activity based on its similarity to known ligand information^9^,^10^.

In SB, molecular docking is widely used, but other techniques are often used in combination, such as homology modeling, which models the target 3D structure when no X-ray structure is available^11^, and molecular dynamics, which searches for a binding site that is not found in the X-ray structure^12^,^13^. In LB, machine learning is used when active ligands and inactive ligands are known^14^,^15^,^16^, and similarity search^17^,^18^ or pharmacophore modeling^19^,^20^,^21^ is used when only active ligands are known. Although these techniques are theoretically expected to be useful for the discovery of promising novel drug candidates, recent studies have shown that the gold standard remains to be established. von Korff *et al.*^22^ conducted a validation study using the Directory of Useful Decoys (DUD)^23^ as a validation set, and in multiple methods of SB and LB, they demonstrated that different methods proposed compounds in different chemical spaces as hit molecules for the same target protein. In other words, the use of multiple different search methods is expected to yield hit molecules in a broader range of chemical space than the use of specific SB or LB methods alone. This study aimed at using multiple CADD methods through open innovation to achieve a level of hit molecule diversity that is not achievable with any particular single method. Therefore, we held a compound proposal contest, in which multiple research groups participated and predicted inhibitors of a target protein. This showed whether collective knowledge based on individual approaches helped to obtain hit compounds from a broad range of chemical space and whether the contest-based approach was effective.

In the contest, a library of 2.2 million commercially available compounds was provided and proposed compounds from each participant group were purchased; these were then tested with an inhibitory assay. Such a large number aimed to simulate a real CADD-oriented procedure. We also chose the tyrosine-protein kinase Yes, a member of the Src family, as a target protein because it has an established inhibitory activity assay protocol. Its inhibition is also clinically important, and participants of the contest could employ either SB and/or LB approaches for this target.

Yes has a multi-domain structure, which consists of SH3 (residues 97–144), SH2 (residues 97–144), and tyrosine kinase (residues 277–526) domains. The Tyr416 residue of the tyrosine kinase domain is phosphorylated during activation of the kinase. Hence, the tyrosine kinase domain located at the C-terminal region of Yes is a direct target for predicting hit compounds. The 3D structures of several kinase proteins have been determined and stored in the Protein Data Bank (PDB)^24^. However, the structure of Yes has not yet been determined (as of April 2014). Our prior-to-the-contest analysis showed that Yes has a high sequence identity with many other protein kinases (e.g., PDBID: 1Y57^25^, 2SRC^26^, 1FMK^27^), of which structures were determined at high resolution. This indicates that homology modeling can be effectively used to obtain the 3D structure of Yes.

On the ligand point of view various open source drug discovery databases such as BindingDB^28^, ChEMBL^29^, DrugBank^30^, and PubChem^31^ contain medicinal chemistry data on a number of drug compounds, active and inactive compounds, and targets. For example, DrugBank is useful for searching known small-compound drugs of Yes, and it contains data on a number of Food and Drug Administration (FDA)-approved small-compound drugs including dasatinib, which has been approved as an anticancer drug that targets mainly Abl and Src family kinases. Kinase SARfari^32^, a satellite database of ChEMBL that contains data on >4000 compounds targeting Src family kinases, is useful for structure–activity relationship (SAR) analysis. Specifically, it contains data on 188 bioactive compounds that inhibit Yes, 30 of which have a half maximal inhibitory concentration (IC_50_) of <50 nM. The availability of bioactivity data aids realistic identification of potential hit compounds.

The compound proposal contest was organized by the Initiative for Parallel Bioinformatics (IPAB). It started on January 7 2014 and ended on March 20 2014. Ten groups participated in the contest. Any groups could participate in the contest if its members agreed that all proposed compounds and methods were to be made public. The participants were asked to propose a prioritized set of 120 compounds. We selected the top 50 compounds from each group and 118 additional compounds via clustering analysis of the submitted compounds, for a total of 600 unique compounds. In an inhibitory activity assay, 24 of the 600 compounds showed inhibition at various ranges and seven were identified as potential hit compounds. Among the potential hits IC_50_ of three compounds with a novel structure were estimated. The salient features of the methods, experimental validations, and potential inhibitors are discussed below.

## Details of the contest

### Compound library

Enamine Ltd provided a collection of approximately 2.2 million small compounds that are commonly used in high-throughput screening (HTS) studies to identify potential hit compounds. The compounds were readily available in the Enamine library; therefore, we used them for screening. Enamine libraries are available at http://www.enamine.net/.

### Computational methods

Different methods have been adopted to identify potential inhibitors of Yes, and they can be roughly classified into the following two categories: the protein structure-based method (SB) and the ligand-based method (LB). Here, we define SB as a docking simulation or a geometric hashing technique that utilizes protein structure. LB involves screening techniques based on structural similarity comparison to known inhibitors or SAR derived from known inhibitors. The comparative analysis of the methods used by the 10 different groups is presented in Table 1. Some groups employed a multi-step approach, where LB was employed to screen the Enamine library and SB was applied to the resultant compounds (denoted by LB → SB). Others used LB and SB simultaneously to screen the Enamine library (denoted by LB&SB).

### Protein structure-based method (Groups 1, 2, 3, 5, 7, 9, and 10)

In this approach, the structure of the target protein is the main focus for identifying potential inhibitors. Initially, the 3D structure of the target protein is obtained via homology modeling using Modeller^33^,^34^,^35^, FAMS^36^,^37^,^38^, etc. Potential inhibitors are then identified using docking, SAR, and molecular dynamics (MD) simulations. The SB method was used by seven of the competing groups, 1, 2, 3, 5, 7, 9, and 10. ***Group 1 (G1)***: Based on a BLAST search of PDB, homologs of Yes that contains ligands were searched, and 25 structures were identified. The Tanimoto indices between the 25 ligands and the Enamine library compounds were calculated, and 1241 compounds with indices >0.55 were chosen for screening. The protein structure used in the docking simulation was created using a template structure with the smallest P-value. Finally, the 1241 compounds were docked using ChooseLD based on FPAScore function. ***Group 2 (G2)***: A series of sequence alignments and binding site investigations were performed to create a protein structure used in subsequent docking simulation and SAR studies. The following three approaches were employed separately: docking, SAR, and similarity comparison with known inhibitors. The docking method used two machine learning systems^39^ based on three docking simulation packages. The SAR model was created using PubChem BioAssay data^40^ (AID 686947) and was applied to the Enamine library. For similarity comparison, a small number of known inhibitors of Yes were selected from the PubChem BioAssay data, and the Enamine library was searched for similar compounds. Finally, the two-dimensional (2D)/3D structures of those compounds as well as their binding poses were compared with those of the native inhibitor found in the target structure. ***Group 3 (G3)***: Known inhibitors were selected from the literature^41^,^42^,^43^,^44^,^45^,^46^ and PubChem BioAssay data^40^ (AID 686946). The Enamine library compounds were screened by similarity search using the known inhibitors. Then, docking simulation of the screened compounds was conducted. The model protein structure was selected based on the docking poses that reproduced those of the known ligands PP2 and dasatinib. Finally, the compounds selected via LB and SB were screened using the pseudo-consensus method^47^. In total, 53 compounds from LB, 53 from SB, and 14 from the pseudo-consensus method were included in the final list. ***Group 5 (G5)***: A multiple-template ligand was modeled using 70 protein-ligand complexes that had a protein sequence identity with Yes of >70%. Proteins and their bound ligands were superimposed by the protein structure alignment program MICAN^48^ against a modeled Yes structure. The Enamine library compounds were compared with the multiple-template model using the geometric hashing technique^49^, where scoring was defined as the number of coincident atoms minus the protein-compound crash penalty, to identify potential hit compounds^50^. ***Group 7 (G7)***: MD simulation and fragment molecular orbital calculation of the Src-dasatinib complex structure were performed to identify residues that interact with dasatinib with high retention or interaction energy, respectively. This information was utilized to define constraints for docking simulation, i.e., to specify specific residues that the docked compounds should interact with. The protein structure for the docking simulation was created using homology modeling. ***Group 9 (G9)***: Data on active ligands were collected from BindingDB^51^, and their physicochemical characteristics were computed and compared with the set of the 2.2 million Enamine library compounds for primary screening. Homology modeling and MD studies were performed to select the best structural orientations, and the resultant eight structures (one homology and seven MD structures) were independently subjected to docking simulation of the screened compounds, active inhibitors, and decoys. The screened compounds with high docking scores were considered only when the protein structures used could supersede those of the active inhibitors and decoys in terms of scores. ***Group 10 (G10)***: The modeled 3D structure of Yes was validated by analyzing its binding poses with known ligands (dasatinib, saracatinib, and bosutinib). These predicted binding poses were captured as a consensus SB pharmacophore model, which was used to screen the Enamine library. To further prioritize the compounds, an enriched substructure filter, which was derived using Src family kinase inhibitors retrieved from BindingDB^51^, was applied to the screened compounds. This list of 2000 potential hit compounds was clustered. Clusters were prioritized after visual inspection, and a representative or the best hit of each cluster was chosen for the final list.

### Ligand-based method (Groups 4, 6, and 8)

In the LB approach, potential hit compounds were primarily identified using the activity data for available kinases. Three of the groups, 4, 6, and 8, used this approach. ***Group 4 (G4)***: The IC_50_s of tyrosine kinase inhibitors were downloaded from Kinase SARfari^52^ and relevant indices (pIC_50_, ligand lipophilic efficiency^53^, binding efficiency index^54^ and surface efficiency index (SEI)^54^) were calculated. Indices were related with physicochemical properties. The experimental indices; physicochemical properties such as hydrophobicity, volume, and pI of the 36 amino acid residues surrounding the ATP binding sites (ABS36)^55^; and compound descriptors were trained with support vector regression, and three models (SEI OETree, SEI MACCS, and SEI OBFP2) were proven to predict experimental values better than other models. These models were applied to the Enamine library to predict the active compounds. It should be noted that Group 4 focused on identifying compounds with good SEI rather than good inhibition activity. ***Group 6 (G6)***: PubChem BioAssay data of 858 compounds^56^ of Yes (AID 686947) were downloaded, and their inhibition rates were normalized at a concentration of 15 μM using linear interpolation between the nearest neighbors of actual measured activities. The activities and a set of molecular descriptors were trained using a random forest model^57^, and the model was utilized for predicting the potential hit compounds. ***Group 8 (G8)***: PubChem BioAssay data of 858 compounds^56^ (AID 686947) of Yes were downloaded. Compounds with an activity <1 μM were defined as active inhibitors, and the rest were classified as inactive. In addition to the inactive inhibitors, some compounds in the Enamine library were defined as inactive to exploit large-scale inactive compounds for training in the SAR model. The SAR model was developed by comparing the activity data with 772 descriptors using the balanced random forest method^57^,^58^ and was applied to the Enamine library to predict active compounds. An imbalance in the numbers of active and inactive compounds was addressed during training^59^.

### Selection of compounds for experimental inhibitory assay

Initially, we selected the topmost 50 compounds from each of the 10 groups to obtain a total of 482 unique compounds. In addition, following cluster analysis, 118 additional compounds were selected using a scoring procedure and manual inspection. We used k-means clustering to classify the compounds into 10 clusters and subsequently computed the similarity score relative to structures of known inhibitors of Src family kinases deposited in ChEMBL using the Tanimoto principle based on MACCS fingerprint^60^. We selected compounds with a maximum similarity score of <0.72 to identify novel inhibitors. We defined a consensus number in each cluster, based on a number of different groups that proposed any compounds to each cluster. From the consensus, we chose 118 compounds, for a total of 600 compounds to be tested by the inhibitory activity assay.

### Experimental procedures

We outsourced the inhibitory activity assay of the proposed compounds to Bienta (http://bienta.net/). Bienta utilized HTS in order to estimate a percentage inhibition rate at 10 μM of each compound. All HTS procedures were performed in accordance with the Promega Technical Manual for ADP-Glo™ Kinase Assay (Fitchburg, WI, USA. Catalog number: V9102). Human recombinant Yes (NCBI reference sequence: NP_005424.1) was purchased from BPS Bioscience (San Diego, CA, USA. Catalog number: 40488). Staurosporine, a well-known pan-kinase inhibitor typically used as a reference in kinase assays, was selected as an active inhibitor for Yes. The copolymer of Glu and Tyr (Glu:Tyr = 4:1, Sigma Aldrich, St. Louis, MO, USA. Catalog number: 81357) was used as a generic tyrosine kinase substrate. The final assay reagent concentrations were 5.5 nM Yes, 0.013 mM ATP, and 0.2 mg/mL substrate. Each compound, at a final concentration of 10 μM, was dispensed in four wells of a 384-well plate. The average of the four values was used to calculate the inhibition rate for each compound. We evaluated each compound for the following three criteria based on the inhibition rate:

- The compound’s inhibition rate was higher than the average inhibition rate of all compounds in the same plate plus three-fold of the standard deviation of inhibition rates in the same plate. In this calculation, the inhibition rates for positive and negative controls were not considered.
- The compound’s inhibition rate was >25% (except for the compounds classified in “A”). This condition was implemented to eliminate false negatives.
- The compound’s inhibition rate was the highest in its group (except for the compounds classified in “B”).

The compounds that identified with any of these criteria proceeded to the secondary assay conducted on one 384-well plate. Each compound was tested in six wells, and the average of the six values was used to determine the percentage inhibition rate. We used an individual inhibition rate of >30% to identify the compounds that could potentially serve as inhibitors to Yes.

## Results and Discussion

### Common compounds identified by different methods

Ten groups each submitted 120 compounds for a total of 1200 compounds. The analysis of the submitted compounds showed that 17 compounds overlapped between two groups, and one compound was the same in three groups. In total, 75% of overlapping compounds were proposed mainly by groups that utilized known ligand information directly or indirectly (G2, G3, G6, and G8). The higher overlapping rate would be attributed to the same information that these LB methods employed, i.e PubChem BioAssay AID 686947. All of the overlapping compounds were selected for the inhibitory activity assay. The behaviors of these compounds were distinct: two were identified as potential hit compounds and the others did not show any inhibition in assay experiments.

### Inhibition rates of selected compounds

We conducted inhibition assay experiments at 10 μM for all of the selected 600 compounds to measure the percentage of inhibition. Among them, 24 compounds satisfied our primary hit conditions and were tested in the secondary assay. The secondary assay was conducted on a single plate, and the results are shown in Table 2. Critical evaluation of these 24 compounds identified seven compounds as probable inhibitors of Yes, and their structures along with their inhibition rates are presented in Table 3. These seven compounds were again tested from their fresh powders to confirm the inhibition rates. Compounds with inhibition rates >50% in the fresh powder assay were Z1546610485 (56.3%, G2, G6, and G8), Z820655914 (89.0%, G5), Z1546616191 (95.4%, G6), Z1157725083 (65.0%, G8), and Z653349554 (66.7%, G10).

Compound Z1546610485 was identified by three groups that employed the LB method (LB or LB&SB in Table 1). The compound is known as gefitinib and is listed in the PubChem Bioactive database^56^ (AID686946 and AID686947) as a tyrosine kinase inhibitor, which may explain why it was independently proposed by three groups. This shows that the LB methods used by these groups could correctly identify a hit compound. On the other hand, Z1546616191, a known tyrosine kinase inhibitor named sunitinib, also listed in the database, was proposed only by G6. It is unclear why the other LB groups did not propose it in their lists of 120 compounds. The number of compounds tested in this contest (a minimum of 50 per group) is insufficient to derive conclusive insight.

According to the PubChem BioAssay data, the inhibition rates of gefitinib and sunitinib at 8.6 μM with 4 nM Yes, 0.1 mM ATP, and 0.3 mg/mL substrate (poly Glu:Tyr = 4:1) were 72.1% and 93.9%, respectively, indicating consistency of our data with the literature. The inhibition rates of a few compounds exceeded that of gefitinib in this study.

Among the 118 compounds that were added by the clustering analysis, there were no potential hit molecules. This might be because the compounds were selected so that their similarity score to known inhibitors was <0.72.

### Chemical space diversity of submitted compounds

**Figure 1:**
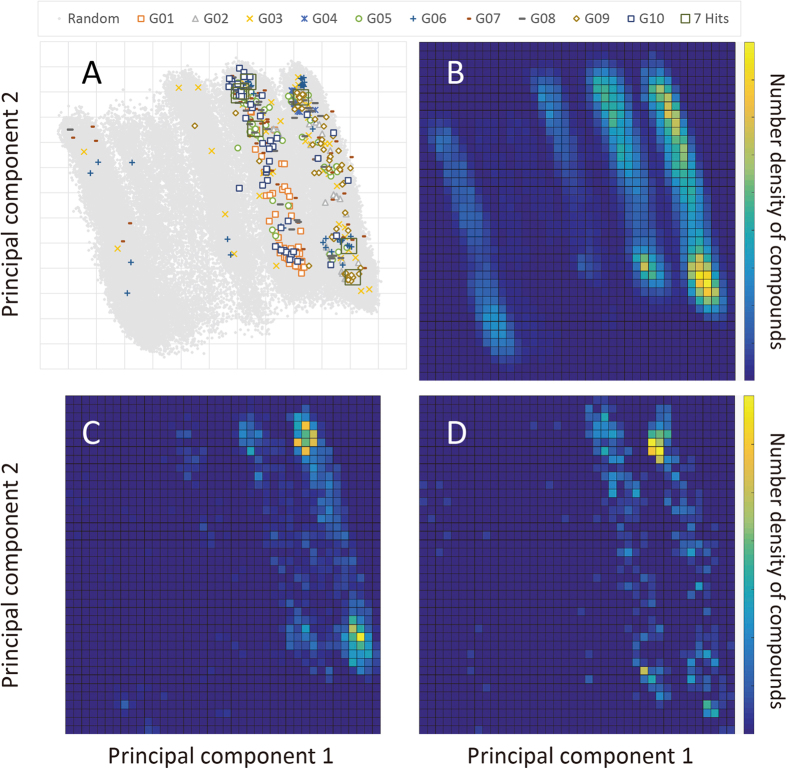
(**A**) Projections of twelve kinds of compounds on the principal component 1 and 2. (**B**) Number of compounds randomly sampled from the compounds library in each grid. (**C**) Number density of the Src known inhibitors. (**D**) Number density of the assayed compounds in this study.

To examine the diversity of submitted compounds we conducted the principal component (PC) analysis of 211546 compounds’ MACCS fingerprint^60^, which were 10% of the compound library and randomly sampled, followed by the projection of the sampled compounds (Random in Fig. 1A), assayed compounds of each group (G1−G10), and the seven potential hits (seven Hits) onto PC1 and PC2. The cumulative variances of PC1 and PC2 were 26% and 50%, respectively, indicating that PC1 and PC2 could well account for the chemical space of the compound library. Figure 1A shows that compounds submitted by the same group show a tendency to gather in the chemical space, i.e., the chemical space covered by compounds submitted only by one group tends to be small. To quantify the coverage, we divided the chemical space into 13 for both PC1 and PC2, as shown in Fig. 1A, and counted a number of grids that contains at least one compound of a group concerned. The coverage numbers for all the groups were as follows: Random: 124, G1: 13, G2: 12, G3: 26, G4: 3, G5: 14, G6: 19, G7: 27, G8: 18, G9: 18, G10: 18, G1−10: 54 (G1−10 contains all the compounds that all the groups submitted), and Known: 89. These values show that the chemical space coverage submitted only by one group tends to be small. On the other hand, the coverage of the merged compounds, G1−10, was comparable to the chemical space of known Src inhibitors. Because the seven potential hits distributed over chemical space that could not be covered only by one group and were different from each other (see structures in Table 3), the contest-based approach can enhance diverse sampling. The coverages of G3 and G7 were relatively high because G3 employed three different approaches and made a compounds list from the three methods. G7 employed the more SB-oriented method that utilized information of lesser known inhibitors.

Figure 1B–D show the number density of the compound library, Src known inhibitors, and assayed compounds, respectively, in the chemical space. The density number map of assayed compounds are not similar to that of the compound library but are similar to Src known inhibitors, indicating that the assayed compounds were not just randomly chosen but enriched toward Src inhibitors.

### Characteristic features of submitted and assayed compounds

**Figure 2:**
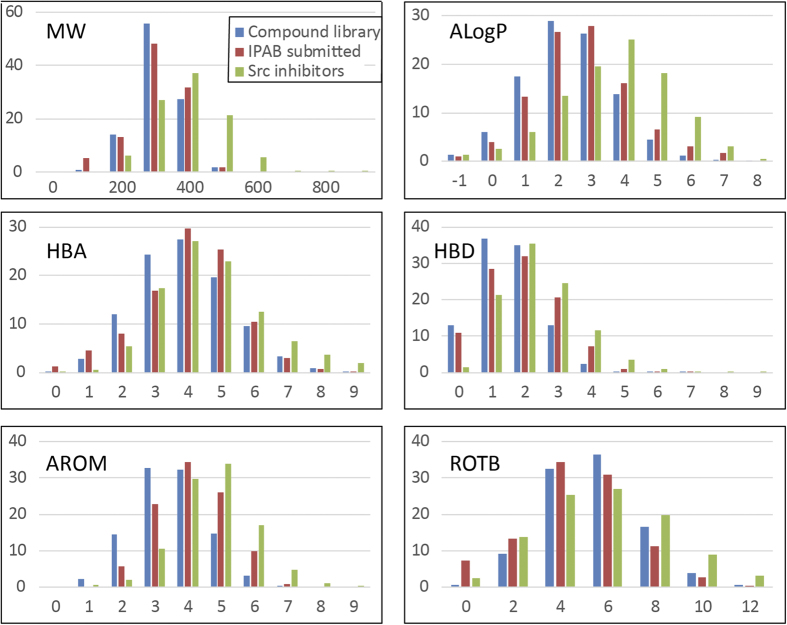
Distribution of compound properties for Enamine library, submitted compounds, and Src family kinase inhibitors.

We analyzed the characteristic features of the 1200 submitted compounds (1180 unique compound structures) using their chemical properties: molecular weight (MW), ALogP, number of hydrogen bond acceptors (HBA), number of hydrogen bond donors (HBD), number of aromatic rings (AROM), and number of rotatable bonds (ROTB) using Canvas Version 2.2.013^61^. In addition, we analyzed the structures of 3528 known Src family kinase inhibitors retrieved from ChEMBL and BindingDB and the randomly selected 211546 structures from the Enamine library for comparison. Figure 2 shows the distribution of the six chemical properties for these three sets of compounds. We observed that for four of the six considered properties, there was a marked difference between the average values of the submitted compounds (AlogP: 3.2, HBA: 3.6, HBD: 1.4, AROM: 3.6) and the Enamine library compounds (AlogP: 2.1, HBA: 3.1, HBD: 1.0, AROM: 2.6), and the properties were biased toward the average values of Src family kinase inhibitors (AlogP: 3.8, HBA: 4.5, HBD: 2.3, AROM: 4.0). Notably, the average ROTB value of the submitted compounds (ROTB: 4.7) was smaller than that of the Enamine library (ROTB: 5.5), which is closer to the average ROTB value of the Src family kinase inhibitors (ROTB: 6.4), although the average ROTB value of the potential hit compounds was 5.7. In addition, the average MW of submitted compounds (MW: 361) was similar to that of the Enamine library (MW: 365), although the average MW of the potential hit compounds was 391, which is closer to the MW of the Src family kinase inhibitors (MW: 455). This analysis suggests that the prediction methods could be improved by considering ROTB and MW. In particular, for docking simulation, special consideration would be necessary when known inhibitors have a large ROTB value, because it is more difficult to cover the conformational space of compounds.

**Figure 3:**
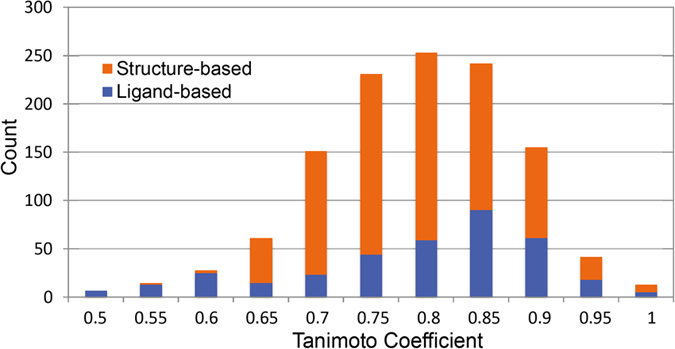
Distribution of similarity scores between submitted compounds and known Src family kinase inhibitors.

Further, we have surveyed the novelty of the submitted compounds. Figure 3 shows a distribution of maximum Tanimoto similarity coefficients for the submitted compounds compared with the known Src family inhibitors. We also measured the difference between the average similarity scores of different approaches to understand the effect of methodologies for selecting compounds. The 10 methods used in the contest are classified into two filter types (see the “Computational methods” section): SB: G1, G2, G3, G5, G7, G9 and G10; and LB: G4, G6 and G8. The average similarity scores for SB and LB are 0.765 and 0.767, respectively, indicating no apparent difference. Because G4′s method was modeled to identify compounds with good SEI, it may affect the average similarity score. The average similarity scores for LB without the inclusion of G4′s compounds is 0.824, indicating that compounds proposed by SB were more novel than those proposed by LB.

### Comparison of different approaches for identifying the potential hit compounds

The systematic comparison of various methods for identifying potential hit compounds can provide insight for a deeper understanding of the concepts of drug design. Among the seven potential hit compounds, six were proposed by groups that either mainly or partly adopted an LB screening process or a ligand template (pharmacophore) derived from known inhibitors. The other compound was proposed by a group that also utilized such information to discriminate a good protein structure for docking from several model structures that are able to discriminate between known active and inactive compounds with respect to docking scores. Therefore, this study indicates that the usage of experimental binding affinity or binding poses is necessary to identify potential inhibitors. This concept reveals the importance of analyzing specific interactions to select potential hit compounds. The application of machine learning techniques helped to map the input features with binding affinity. Further, SB methods combined with pharmacophore modeling and docking could be useful in identifying potential hit compounds. Overall, the comparison of methods indicates the importance of balancing between LB and SB methods to identify inhibitors. Furthermore, we observed that the inclusion of visualization and detailed analysis are important for identifying potential hit compounds. With respect to speed, LB methods are faster than SB models, and machine learning techniques could aid successful prediction.

As for the novelty of the potential hit compounds, the LB methods identified compounds similar to the known Src family kinase inhibitors (e.g., similarity scores of Z1157725083, Z1546616191, and Z1546610485 relative to known inhibitors were 0.80, 1.0, and 1.0, respectively). On the other hand, the SB methods predicted compounds with relatively lower similarity to the known Src family kinase inhibitors (e.g., similarity scores of Z820655914, Z126204226, and Z653349554 were 0.75, 0.77, and 0.79, respectively). When novelty is of interest, an SB method with the aid of known inhibitor information and/or docking poses is a good choice.

In addition, we calculated two different ligand efficiency indices: inhibition rate (%) divided by MW or topological polar surface area (TPSA), as shown in the Supporting Information (Supplementary Fig. S1). Two compounds (Z1095352660 and Z993990690) proposed by an LB approach (G4) are plotted in the upper-left corner of Supplementary Fig. S1. These compounds are small (MW of 151 and 255 for Z1095352660 and Z993990690, respectively) but have relatively high ligand efficiencies compared to their sizes (inhibition rate/MW: 0.26 and 0.10, respectively; inhibition rate/TPSA: 2.5 and 2.0, respectively). G4′s method focused on SEI and successfully identified compounds with high ligand efficiency comparable to those of known inhibitors.

### Comparison between the potential hit and negative compounds

**Figure 4:**
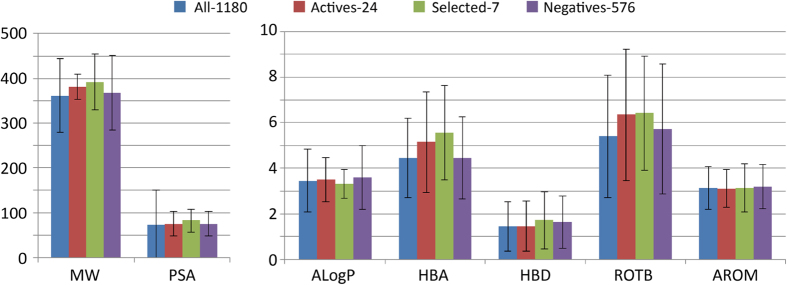
Physicochemical characteristics of all the 1180 submitted, 24 active, 7 selected, and 574 negative compounds.

The list of compounds that did not show any inhibition of Yes is presented in Supplementary Table S1. These compounds can be used as decoys for docking and other studies. We have analyzed the characteristic features of the seven potential hit compounds and performed a comparison with the negative compounds. The physicochemical features of all 1180 proposed compounds, 24 active compounds, and seven selected compounds, along with the 574 decoys, are shown in Fig. 4. Because there are no significant differences between the selected compounds and the rest of the submitted compounds, the negative compounds could be good decoys and may be helpful to further refine active inhibitors.

### The strategy of using different approaches together

The contest based approach is the outcome of ten individual methods (Table 1), which are independent to each other on various perspectives: (i) different templates to obtain the target in SB approach, (ii) database of actives and decoys in LB method, (iii) variations in software packages for identifying the hits and docking and (iv) scoring procedures for ranking the hit compounds. Although the main objective of each method is to identify the lead compounds by covering a large chemical space and utilizing standard procedures none of them is able to identify all the hit compounds, which have been observed experimentally. We anticipated that all the methods could identify few hit compounds and most of them are not overlapping with each other. Hence, we have used the strategy of collecting the top ranked compounds in each method for verifying the hit compounds using experimental techniques.

### The advantages of using different approaches together

Each prediction method utilized advanced techniques and reliable procedures reported in the literature for identifying potential hit compounds. The overlapping compounds are minimal among different methods and all methods provided diverse list of compounds with a strong basis for understanding the activity. Further, no single method is efficient to identify the hit compounds and it is not possible for a single group to perform all computational methods. The outcome of each method is complimenting with each other and hence the combination of methods could help to identify the hit compounds realistically. Interestingly, the hit compounds identified by experiments have been proposed by different groups participated in the contest. The contest based approach made it possible to narrow down the experiments from 2.2 million to 600 compounds and 24 of them are identified as hits.

### Suggestions for future based on the experience gained in this contest

The outcome of the contest based approach provide several insights for future directions: (i) comparative performance of structure based and ligand based approaches for identifying the hits, (ii) list of actives and decoys for the target cYes kinase, which could be used to refine the methods and validating new methods, (iii) probable interaction and binding modes for target based drug design, (iv) utilizing efficient, reliable and wide range of information for identifying lead compounds and (v) combination of methods to identify and rank potential compounds. Looking back into known experimental data on several ligands it is possible to predict additional compounds with better affinity and understand the mechanism.

## Conclusions

We conducted a contest-based approach to identify various inhibitors of the tyrosine-protein kinase Yes. In total, 10 groups participated in the contest and tackled the challenge using their own methods. The proposed compounds from all the groups collectively had a more diverse chemical space than compounds proposed only by each group, indicating that a contest-based approach can supply the early stage of drug discovery with various initial inhibitors. The contest was also successful in identifying 24 compounds with inhibition activity and seven potential hit compounds. The IC_50_ evaluation of Z820655914, Z653349554, and Z1157725083 by the 8-point curve showed that the values of Z820655914 and Z1157725083 were >100 μM. The values for Z653349554 suggested that it had been reacted with a reagent. The potential hit compounds can be further considered for the next phase of drug design. Our study revealed that using information about known inhibitors or their docking poses was necessary for both the LB and the SB approaches.

## Additional Information

**How to cite this article**: Chiba, S. *et al.* Identification of potential inhibitors based on compound proposal contest: Tyrosine-protein kinase Yes as a target. *Sci. Rep.* **5**, 17209; doi: 10.1038/srep17209 (2015).

## Supplementary Material

Supplementary Information

## Figures and Tables

**Table 1:** Details of Yes structure and processing methods of Enamine library by different groups to select 120 compounds.

Group ID	Modeling of Yes structure		Processing method of Enamine Library		
	3D structure prediction methods/tools	Template(s) PDB ID	Filter class	Actives^a^	Decoys
1	FAMS	1Y57	LB → SB^b^	Co-crystalized ligands in PDB	—
2	Prime	2SRC	LB^a^ LB&SB	PubChem BioAssay (AID 686947)	—
3	Modeller	1Y57	LB^a^ → SB LB^a^ LB^a^&SB	Pubchem BioAssay (AID686946)	—
4	—	—	LB^c^	Kinase SARfari	—
5	Modeller	Close homologs	LB&SB	Co-crystalized ligands in PDB	—
6	—	—	LB^a^	Pubchem BioAssay (AID686947)	—
7	Prime	3G5D	SB	Co-crystalized dasatinib in PDB (3G5D)	—
8	—	—	LB^a^	Pubchem (AID686947)	—
9	Prime	2SRC	SB	BindingDB	DUD-E
10	Modeller	1FMK	SB → LB^a^	Dasatinib, bosutinib & saracatinib BindingDB < 500 nM	BindingDB ( > 500 nM)

^a^Yes specific/Src kinase family inhibitors reported using experimental methods.

^b^Ligands collected from cocrystallized structures that show more sequence similarity with Yes (25 ligands for group 1 and 70 for group 2).

^c^Descriptor of residue surrounding ATP binding pocket was also used. LB, SB and ML denote ligand based, structure base and machine learning approaches used for initial filtering of 2.2 million Enamine library compounds.

**Table 2:** Activity details of the 24 compounds that show minimum 25% inhibition at a concentration of 10 μM.

Compound ID^a^	Group ID	Inhibition rate^b^%	Primary assay			Secondary assay	
			Standard deviation %	Plate criterion^c^ %	Primary hit condition	Inhibition rate^d^ %	coefficient of variance %
Z1139201021	1	26.6	3.6	40.7	C	17.9	4.5
Z1546610485	2	64.6	3.6	21.1	A	62.2	7.0
Z118332804	2	28.7	6.2	31.8	C	1.6	20.1
Z235987838	3	28.8	13	35.9	C	7.2	23.0
Z1095352660	4	38.9	10.4	40.7	A	19.0	8.8
Z993990690	4	26	10.9	40.7	C	11.0	11.0
Z240877358	5	25.1	12.4	35.9	C	19.5	11.7
Z56829275	5	20.6	10.2	22.1	A	20.9	7.9
Z820655914	5	37.6	2.8	36.9	A	39.0	28.0
Z1546610485	6	64.6	3.6	21.1	A	62.2	7.0
Z1546616191	6	95.6	0.6	21.1	A	98.7	14.6
Z31233162	6	31.1	6	31.8	A	6.4	6.4
Z230779338	6	27.4	7.8	35.9	C	−0.5	15.3
Z56864857	6	22.2	7.7	22.1	A	8.4	12.2
Z279622612	6	57.4	43.4	37.2	A	22.8	14.1
Z17897344	7	26.6	4.8	35.9	C	23.3	7.1
Z1157725083	8	20.2	10.8	40.7	C	29.3	8.4
Z1546610485	8	64.6	3.6	21.1	A	62.2	7.0
Z295506072	9	24.9	5.4	40.7	C	8.8	10.8
Z126204226	9	32.9	11.1	35.9	B	26.9	8.9
Z356233398	9	39	45.6	22.1	A	24.8	11.3
Z254598624	9	31.3	11.7	40.7	B	13.0	13.8
Z1338036236	9	31.2	7.7	40.7	B	17.7	22.5
Z1024444840	10	29	9.5	37.2	C	44.9	13.7
Z653349554	10	50.5	1.4	36.9	A	86.9	14.4
Z728752856	10	53.1	4.7	22.1	A	18.4	8.1

^a^Defined by Enamine Ltd.

^b^Average of four rates obtained in primary assay.

^c^Sum of inhibition-rate average on the plate and the threefold of the standard deviation.

^d^Average of six rates.

**Table 3:**
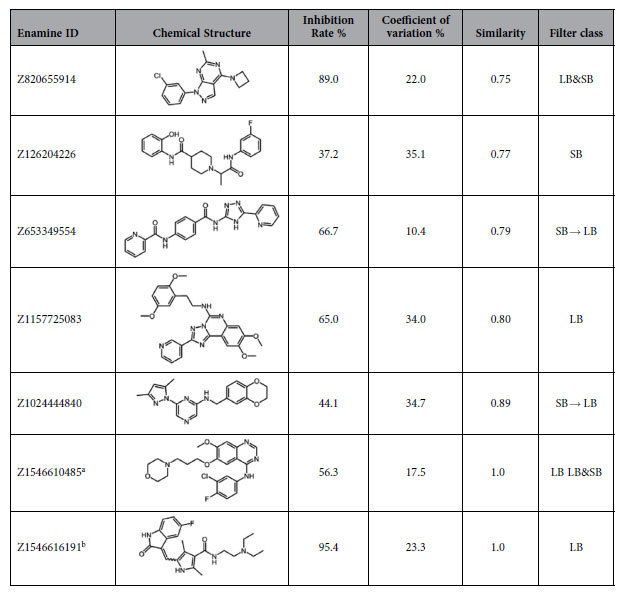
Potential hit compounds in validation assay (from fresh powder).

^a^The compound is known as gefitinib.

^b^The compound is known as sunitinib.
