# Supplementary material for: Identification of potential inhibitors based on compound proposal contest: Tyrosine-protein kinase Yes as a target
Source: Sci Rep. 2015 Nov 26;5:17209. doi: 10.1038/srep17209 (PMC4660442; doi:10.1038/srep17209)
Supplement: Supplementary Information [file srep17209-s1.pdf]

Supplementary Materials for:

Identification of potential inhibitors based on compound proposal contest: Tyrosine-protein kinase Yes as a target

Shuntaro Chiba[1], Kazuyoshi Ikeda[2], Takashi Ishida[1,3], M. Michael Gromiha[4],Y-h. Taguchi[5], Mitsuo Iwadate[6], Hideaki Umeyama[6], Kun-Yi Hsin[7], Hiroaki Kitano[7,8,9], Kazuki Yamamoto[10], Nobuyoshi Sugaya[11], Koya Kato[12], Tatsuya Okuno[13], George Chikenji[12], Masahiro Mochizuki[14], Nobuaki Yasuo[3], Ryunosuke Yoshino[15,16], Keisuke Yanagisawa[3], Tomohiro Ban[3], Reiji Teramoto[17], Chandrasekaran Ramakrishnan[4], A. Mary Thangakani[18], D. Velmurugan[18], , Philip Prathipati[19], Junichi Ito[19], Yuko Tsuchiya[19], Kenji Mizuguchi[19], Teruki Honma[20], Takatsugu Hirokawa[21,22], Yutaka Akiyama[1,3,21,22], Masakazu Sekijima[1,3,15,22*]

[1]Education Academy of Computational Life Sciences (ACLS), Tokyo Institute of Technology, 4259 Nagatsutacho, Midori-ku, Yokohama 226-8501 Japan

[2]Level Five Co. Ltd., Shiodome Shibarikyu Bldg., 1-2-3 Kaigan, Minato-ku, Tokyo 105-0022, Japan

[3]Department of Computer Science, Tokyo Institute of Technology, 2-12-1, Ookayama, Meguro-ku, Tokyo 152-8550 Japan

[4]Department of Biotechnology, Bhupat and Jyoti Mehta School of Biosciences, Indian Institute of Technology Madras, Chennai 600 036, Tamilnadu, India[5]Department of Physics, Chuo University, 1-13-27 Kasuga, Bunkyo-ku, Tokyo 112-8551, Japan

[6]Department of Biological Sciences, Chuo University, 1-13-27 Kasuga, Bunkyo-ku, Tokyo 112-8551, Japan

[7]Okinawa Institute of Science and Technology Graduate University, 1919-1 Tancha, Onna-son, Kunigami, Okinawa 904-0495 Japan

[8]The Systems Biology Research Institute, Falcon Building 5F, 5-6-9 Shirokanedai, Minato-ku, Tokyo 108-0071 Japan

[9]Center for Integrative Medical Sciences, RIKEN,1-7-22 Suehiro-cho, Tsurumi-ku,Yokohama City, Kanagawa, 230-0045, Japan

[10]Research Center for Advanced Science and Technology, The University of Tokyo, 4-6-1 Komaba, Meguro-ku, Tokyo 153-8904 Japan

[11]PharmaDesign Inc., 2-19-8, Hatchobori, Chuo-ku, Tokyo 104-0032 Japan

[12]Department of Computational Science and Engineering, Nagoya University, Furocho, Chikusa, Nagoya 464-8603, Japan

[13]Division of Neurogenetics, Nagoya University Graduate School of Medicine, 65

Tsurumai, Showa-ku, Nagoya 466-8550, Japan
[14]Information and Mathematical Science and Bioinformatics Co., Ltd., Level 6 OWL TOWER, 4-21-1 Higashi-Ikebukuro, Toshima-ku, Tokyo 170-0013 Japan
[15]Global Scientific Information and Computing Center, Tokyo Institute of Technology 2-12-1, Ookayama, Meguro-ku, Tokyo 152-8550 Japan
[16]Department of Biotechnology, The University of Tokyo, 1-1-1 Yayoi, Nunkyo-ku, Tokyo, 113-8657
[17]Forerunner Pharma Research, Co., Ltd., Yokohama Bio Industry Center, 1-6 Shuehiro-cho, Tsurumi-ku, Yokohama 230-0045 Japan
[18]Centre of Advanced Study in Crystallography and Biophysics and Bioinformatics Infrastructure Facility (DBT Funded), University of Madras, Chennai 600025, Tamilnadu, India
[19]National Institute of Biomedical Innovation, Health and Nutrition, 7-6-8 Saito-Asagi, Ibaraki, Osaka 567-008 Japan
[20]Center for Life Science Technologies, RIKEN, 6-7-3 Minatojima-minamimachi, Chuo-ku, Kobe-shi, Hyogo 650-0047 Japan
[21]Molecular Profiling Research Center for Drug Discovery, National Institute of Advanced Industrial Science and Technology, 2-4-7 Aomi,Koto-ku,Tokyo,135-0064, Japan
[22]Initiative for Parallel Bioinformatics, Level 14 Hibiya Central Building, 1-2-9 Nishi-Shimbashi Minato-Ku, Tokyo 105-0003 Japan

*Corresponding author

Table S1. Inhibition rates of negative compounds in the primary assay.

ID	SMILES	Inhibition rate of orimary assay at 10 uM	Standard deviation of four assay %
Z961129172	C#CCNC(=O)C1CCN(CC1)c2ncnc(c23)n(C)nc3	6.9	9.1
Z820668426	C1CCCC1NC(=O)CC2CCN(CC2)c3ncnc(c34)n(C)nc4	23.2	5.5
Z1127122422	c1nn(C)c(c12)ncnc2N3CCCN(CC3)c4ncccn4	11.2	15.3
Z57385379	c1cc(C)ccc1Cn(nc2)c(c23)ncnc3N(CC4)CCC4C	9.8	7.9
Z646400624	c1nn(C)c(c12)ncnc2Nc(cc3F)cc(F)c3N4CCCC4	-7.7	13
Z234484874	c1cccc(Cl)c1Cn(nc2)c(c23)ncnc3N(CC4)CCC4C	11.3	8.3
Z58286917	c1ccccc1Cn(nc2)c(c23)ncnc3N4CCCC4	11.1	5.9
Z1357254472	COC(=O)Cn(nc1)c(c12)ncnc2N(C3)CCC34CCCCCC4	-4.8	11.2
Z994868030	O1CCOC12CCN(CC2)c(nc(c34)n(C(C)(C)C)nc3)nc4N(CC5)CCC56OCCO6	13.6	9.2
Z1172235384	c1cc(Cl)ccc1Cn(nc2)c(c23)ncnc3-n4ccnc4	-2	13.5
Z1172235373	c1ccccc1Cn(nc2)c(c23)ncnc3-n4ccnc4	-5.6	5.2
Z228914588	CCCCNC(=O)CN1CCN(CC1)c2ncnc(c23)n(C)nc3	10.9	5.2
Z229001636	COc(cc1)c(OC)cc1CCCN(C)c2ncnc(c23)n(C)nc3	19.4	4.1

Z1449882931	CC(C)(C)n(nc1)c(c12)nc(C)nc2N(CCO)CCOCC(F)(F)F	-0.6	14.3
Z1262402580	CN(C)CC(C)(C)C(C)Nc1ncnc(c12)n(nc2)CC(=O)OC	3	4.1
Z354320796	FC(F)n1ccnc1CN(C)c2ncnc(c23)n(C)nc3	26.2	7.2
Z751560976	CC(C)(C)OC(=O)NC1CCCN(C1)c2ncnc(c23)n(C)nc3	15.2	5.2
Z1449894301	CC(C)(C)n(nc1)c(c12)nc(C)nc2NCc3ccnc(c34)cccc4	-9.3	6.4
Z1419966801	COC(=O)Cn(nc1)c(c12)ncnc2N3CCCCC3CCC(C)C	-4.5	12.3
Z838123396	c1ccccc1CN(CC2)CCC2CNc3ncnc(c34)n(C)nc4	7	3
Z1528987212	CC(C)(C)n(nc1)c(c12)ncnc2N(C)Cc3ccc(F)cc3	-10.4	9.6
Z57075568	c1cc(Cl)ccc1Cn(nc2)c(c23)ncnc3N4CCCC4	20.1	11.3
Z1124161607	CCN(CC)C(=O)C1CCCN1c2ncnc(c23)n(C)nc3	10.7	13.6
Z257372954	C1CCCC1C(=O)N2CCN(CC2)c3ncnc(c34)n(C)nc4	3.5	12.2
Z645932702	C1CCCN1C(=O)C(=O)N2CCN(CC2)c3ncnc(c34)n(C)nc4	10.9	9
Z1170427283	COCC(=O)N1CCCCCC1C2CCN(CC2)c3ncnc(c34)n(C)nc4	4.3	10.7
Z223835066	C1CC=CC1CC(=O)N2CCN(CC2)c3ncnc(c34)n(C)nc4	-17.7	21.9
Z223820614	CCCNC(=O)CN1CCN(CC1)c2ncnc(c23)n(C)nc3	16.3	9
Z354311120	CC(C)NC(=O)CN1CCCN(CC1)c2ncnc(c23)n(C)nc3	1.9	6.5
Z229000416	c1cccc(F)c1OCCN(C)c2ncnc(c23)n(C)nc3	-7.5	17.7
Z57463350	c1cc(Cl)ccc1Cn(nc2)c(c23)ncnc3N(CC4)CCC4C	10.8	9.2

Z22900 8208	c1ccccc1OCCCNc2ncnc(c23)n(C)nc3	25.7	6.1
Z32869 3906	CNC(=O)c1ccc(cc1)CN(C2CC2)c3ncnc(c34)n(C)nc4	24.7	16.4
Z22862 9416	c1cccc(Cl)c1Cn(nc2)c(c23)ncnc3N(C4)CC(C)CC4C	6.4	5.2
Z14086 93759	COC(=O)Cn(nc1)c(c12)ncnc2N(C)C(C3CC)CCCC3	13.5	14.7
Z13484 40370	COC(=O)Cn(nc1)c(c12)ncnc2NC(C(C)C)CO	5.1	6.4
Z64659 9014	c1cccc(OC)c1OCCN(C)c2ncnc(c23)n(C)nc3	23.7	12.9
Z24167 0488	CCNC(=O)CN1CCN(CC1)c2ncnc(c23)n(C)nc3	6.6	13.8
Z11722 35399	c1cc(C)ccc1Cn(nc2)c(c23)ncnc3-n4ccnc4	1.9	11.9
Z15290 33298	CC(C)(C)n(nc1)c(c12)ncnc2N(CC3C)CCN3CCOCC	26.4	3.1
Z72908 1474	CCC(=O)N1CCCN(CC1)c2ncnc(c23)n(C)nc3	18.7	8.7
Z57170 044	c1cc(Cl)ccc1Cn(nc2)c(c23)ncnc3N4CCCCC4	13.7	8.7
Z90963 6296	c1cccc(c12)n(CC)c(n2)CN(C)c3ncnc(c34)n(C)nc4	21.2	8.1
Z11375 93738	OCCCSCCNc1ncnc(c12)n(C)nc2	3.6	11.3
Z13798 75764	COC(=O)Cn(nc1)c(c12)ncnc2N(C(C)C)CCCCC	4.8	4.3
Z22382 1658	CCN(CC)C(=O)CN1CCN(CC1)c2ncnc(c23)n(C)nc3	5.3	8.5
Z80825 0054	c1cc(F)ccc1CCN(C)c2ncnc(c23)n(C)nc3	11.5	11.3
Z99486 6276	C1CCCN1C(=O)CN(C)c2nc(nc(c23)n(C(C)(C)C)nc3)N(C)CC(=O)N4CCCC4	24.8	7.2
Z22873 4336	c1ccccc1OCCN(C)c2ncnc(c23)n(C)nc3	14.4	7.5

Z1640890828	CC(C)(C)n(nc1)c(c12)ncnc2NC(C)Cc3cc(O)c(O)cc3	-18.9	12.6
Z1501480425	OCCNCCNc1ccc(NCCNCCO)c(c12)C(=O)c3c(C2=O)c(O)ccc3O	-5.9	7.5
Z1546624232	C1CN(C)CCN1Cc(cc2)ccc2C(=O)Nc(cc3)cc(c3C)Nc(n4)nccc4-c5cccnc5	4.6	10.4
Z56227192	c1cccc(O)c1C(=O)c(c2)cnc(c23)n(CCO)nc3-c4ccccc4	26.1	7.5
Z244373888	COc(cc1)c(OC)cc1C(N2CCOCC2)CNc3nc(-c4cccnc4)nc(c35)cccc5	4.1	15.2
Z55864190	c1c(Cl)ccc(O)c1C(=O)c(c2)cnc(c23)n(CCO)nc3C	12.7	3
Z55864153	c1c(F)ccc(O)c1C(=O)c(c2)cnc(c23)n(CCO)nc3C	-5	7.6
Z55864338	c1c(Cl)cc(Cl)c(O)c1C(=O)c(c2)cnc(c23)n(CCO)nc3C	24	4.9
Z56811117	Cc(c1)ccc(c1C)NC(=O)c2cc(ccc2)Nc(nc(c34)ccc(Cl)c4)nc3-c5ccccc5	15.8	2.8
Z220617850	c1ccc(Cl)cc1NC(=O)c2ccc(cc2)Nc(nc(c34)cccc4)nc3-c5ccccc5	-4.8	8.5
Z57380492	c1ccccc1NC(=O)c2cc(ccc2)Nc(nc(c34)ccc(Br)c4)nc3-c5ccccc5	21.8	8.5
Z220617840	c1cc(O)ccc1NC(=O)c2cc(ccc2)Nc(nc(c34)ccc(C)c4)nc3-c5ccccc5	2.5	8.3
Z1412755282	c1ccc(C)c(c1C)NC(=O)c2ccc(cc2)Nc(nc(c34)cc(Cl)cc4)nc3-c5ccccc5	4.9	10.2
Z57110409	c1ccccc1NC(=O)c2ccc(cc2)Nc(nc(c34)ccc(C)c4)nc3-c5ccccc5	9.9	7.7
Z56811126	c1ccccc1NC(=O)c2ccc(cc2)Nc(nc(c34)ccc(Br)c4)nc3-c5ccccc5	10.4	3.5
Z1551844014	c1ccc(OC)cc1NC(=O)c2ccc(cc2)Nc(nc(c34)ccc(C)c4)nc3-c5ccccc5	9.7	7.8
Z220617848	Cc1ccc(cc1)NC(=O)c2ccc(cc2)Nc(nc(c34)ccc(C)c4)nc3-c5ccccc5	-5.4	5.6
Z56808888	c1cc(Cl)ccc1NC(=O)c2ccc(cc2)Nc(nc(c34)ccc(Cl)c4)nc3-c5ccccc5	-12.1	6

Z1540085909	c1cccc(Cl)c1N(CC2)CCN2C(=O)c3cc(ccc3)Nc(nc(c45)ccc(Cl)c5)nc4-c6ccccc6	9.6	5.6
Z1409331242	c1cccc(Cl)c1N(CC2)CCN2C(=O)c3ccc(cc3)Nc(nc(c45)ccc(Br)c5)nc4-c6ccccc6	6	21.5
Z1413767126	c1cccc(Cl)c1N(CC2)CCN2C(=O)c3ccc(cc3)Nc(nc(c45)cc(Cl)cc5)nc4-c6ccccc6	-2.9	11.7
Z57380489	c1ccccc1N(CC2)CCN2C(=O)c3ccc(cc3)Nc(nc(c45)ccc(Cl)c5)nc4-c6ccccc6	11.8	6
Z56832109	CCCOc(cc1)ccc1-c(nc(c23)cccc3)nc2Nc(c4)ccc(Cl)c4Cl	2.6	3.2
Z90250646	c1cccc(C2=O)c1C(=O)c(c23)cccc3NC(=O)CN4CCN(CC4)Cc5cccc(c56)cccc6	-6.2	6.1
Z57110492	C1CN(C)CCN1C(=O)c2ccc(cc2)Nc(nc(c34)ccc(Cl)c4)nc3-c5ccccc5	17.6	8.1
Z56844693	COc(cc1)ccc1Nc2ncnc(c23)cccc3	12	9.2
Z270735308	COc(cc1)ccc1Nc2ncnc(c23)ccc(Cl)c3	9.4	13.5
Z281802592	COc1c(OC)ccc(c1)Nc2ncnc(c23)cccc3	3.6	9.7
Z57081336	CCOc(cc1)ccc1Nc2nc(C)nc(c23)cccc3	10.4	7.9
Z397858992	COCCOc(cc1)ccc1Nc2ncnc(c23)cccc3	1.5	3.9
Z228759408	c1cc(Cl)cc(c12)ncnc2Nc(c3)ccc(c34)OCCCO4	23	7.4
Z118332870	c1cccc(F)c1OCCNc2ncnc(c23)cc(OC)c(c3)OC	25.3	10.1
Z118527078	c1cccc(c12)OCC(O2)CNc3ncnc(c34)cc(Cl)cc4	26.9	7.4
Z46381324	c1ccc(OC)c(OC)c1CN(C)CC(=O)Nc2cccc(c23)C(=O)c4c(C3=O)cccc4	2.3	7.7
Z46444703	c1cccc(Cl)c1C(C)NCC(=O)Nc2cccc(c23)C(=O)c4c(C3=O)cccc4	3.7	10.7
Z46313150	c1ccc(OC)cc1CN(C)CC(=O)Nc2cccc(c23)C(=O)c4c(C3=O)cccc4	-0.5	17.1

Z46372242	COc(cc1)c(OC)cc1CN(C)CC(=O)Nc2cccc(c23)C(=O)c4c(C3=O)cccc4	-1.1	6.7
Z57976235	c1c(Cl)ccc(O)c1C(=O)c(cn2)cn(c23)nc(n3)CCCO	7.5	13.4
Z57975467	c1c(F)ccc(O)c1C(=O)c(cn2)cn(c23)nc(n3)CCCO	22.7	7.1
Z46320490	CCOc(cc1)c(OC)cc1CN(C)CC(=O)Nc2cccc(c23)C(=O)c4c(C3=O)cccc4	17.1	14.8
Z163420324	CCOc(cc1)c(OC)cc1CN(CC)CC(=O)Nc2cccc(c23)C(=O)c4c(C3=O)cccc4	16.3	16.7
Z46421033	Cc1c(C)cccc1NC(=O)CN(C)CC(=O)Nc2cccc(c23)C(=O)c4c(C3=O)cccc4	26.7	3.8
Z46504318	c1cccc(c1C)N(CC2)CCN2CC(=O)Nc3cccc(c34)C(=O)c5c(C4=O)cccc5	3.6	12.3
Z46200293	c1cccc(Cl)c1N(CC2)CCN2CC(=O)Nc3cccc(c34)C(=O)c5c(C4=O)cccc5	2.5	8.6
Z46223556	Cc1c(C)cccc1N(CC2)CCN2CC(=O)Nc3cccc(c34)C(=O)c5c(C4=O)cccc5	17.3	6.5
Z44516332	c1cccc(O)c1N(CC2)CCN2C(C)C(=O)Nc3cccc(c34)C(=O)c5c(C4=O)cccc5	5.2	6
Z46203278	c1ccc(Cl)cc1N(CC2)CCN2CC(=O)Nc3cccc(c34)C(=O)c5c(C4=O)cccc5	13.2	5.3
Z46174104	c1ccccc1N(CC2)CCN2CC(=O)Nc3cccc(c34)C(=O)c5c(C4=O)cccc5	15.4	4.6
Z46211489	n1cccnc1N(CC2)CCN2CC(=O)Nc3cccc(c34)C(=O)c5c(C4=O)cccc5	15.9	6.7
Z56759010	ClCC(=O)Nc1cccc(c12)C(=O)c3c(C2=O)cccc3	17	6.1
Z1230097675	CC(C)(O)COc(cc1)ccc1Nc2cc(Cl)nc(n2)N	17.7	6.9
Z1331548892	c1cc(C)nc(c12)ncc(c2O)C(=O)NCC(CO)Cc3c(F)ccc(F)c3	13.9	4
Z31253513	COc1c(Cl)cc(cc1)Nc2nc(-c3cccnc3)nc(c24)cccc4	0.8	5.9
Z239617942	COc(c1)c(OC)cc(c1C)Nc2nc(-c3ccncc3)nc(c24)cccc4	1.9	7.4

Z31184275	c1cccc(c12)nc(-c3cccnc3)nc2Nc(c4)ccc(c45)OCCCO5	0.8	6.8
Z31070725	c1ccc(OC)cc1C(N2CCCC2)CNc3nc(-c4cccnc4)nc(c35)cccc5	22.8	6.8
Z25060862	c1cccc(c12)nc(c(c2C)C(=O)OCC)COC(=O)C34CC5(O)CC(C3)CC(C4)C5	-0.5	19.8
Z19211876	C1C(C2)CC(C3)CC2CC13NC(=O)COC(=O)c4c(CC)c(CCC)nc(c45)cccc5	2.3	3.3
Z26510790	C1C(C2)CC(C3)CC2CC13C(C)NC(=O)c(c4)ccc(c45)C(=O)N(C5=O)Cc6ccncc6	-1.8	5.5
Z48754256	C1CCCCC1NC(=O)Cn(\c(s2)=C\C(=O)C(C)(C)C)c(=O)\c2=C\c3cccc(c34)cccc4	-7.9	5.1
Z18260190	COc(c1)ccc(OC)c1\C=C\C(=O)OCC(=O)Nc(cccc2)c2C(=O)NC3CCCC3	14.4	5.9
Z25878227	N#Cc1c(=O)n(C2CC2)cc(c1)C(=O)c3c(ccc(C)c3)OCC(=O)Nc(c4)ccc(c45)CCC5	6.9	5.3
Z89960427	Cc1csc(=O)n1CCC(=O)OCc2csc(n2)Nc3ccc(cc3)OC	19.7	4.7
Z217747250	C1COc(c12)ccc(c2)C(=O)CN(C3=O)C(=O)c(c34)cccc4	3.5	9.7
Z942116646	c1cccc(c12)[nH]c(c2)CNC(=O)c3c(=O)cc(C)n(n3)-c(cc4C(F)(F)F)ccc4	0.2	16.2
Z56951930	Cc1ccc(cc1)-c2nn(c(c23)nc(C)cc3O)-c4ccccc4	24.2	6.4
Z221050600	c1ccccc1-c(cco2)c2C(=O)NCCn(c(n3)C)c(c34)cccc4	0.4	12.3
Z90787213	c1cccc(c12)n(CCC)c(n2)NC(=O)c3c(C)nc(s3)-c4cccs4	6.9	5.7
Z167050178	Cc1c([N+]([O-])=O)cc(cc1)NC(=O)c2c(C)nc(cc2)-c3ccccc3	15.2	5.8
Z56915658	c1cccc(c12)nc(-c3ccncc3)cc2C(=O)N(c4oc(-c5ccco5)c(c4C#N)-c6ccco6)C(=O)c7cc(-c8ccncc8)nc(c79)cccc9	13.1	10
Z104342398	C1CCCCC1NC(=O)c2cc(ccc2)NC3=C(Cl)C(=O)N(C3=O)Cc4ccccc4	11.2	5.7
Z111581602	c1cccc(c12)oc(c2)C(=O)Nc(c3)ccc(Cl)c3C(=O)OC	-8.3	7.1

Z144323220	CC1C(C)Oc(c12)c(ccc2)C(=O)Nc(ccc3)cc3C(=O)Nc(c4C)c(=O)n(n4C)-c5ccccc5	13.9	9.9
Z385259374	Cc(n1)scc1CSc(cc2)ccc2C(=O)Nc(cc3)ccc3CN(CC4)CCN4CC	-18.4	10.4
Z44306217	c1cccc(c12)NC(=O)C\2=C\c3csc(n3)-c4ccc(Cl)cc4	-14.4	21.7
Z99494490	c1cccc(c12)nc(-c3cccs3)cc2C(=O)N(CC4)CCC4C(=O)Nc(c5)ccc(c56)OCO6	-5.2	4.9
Z821336010	CNC(=O)c1ccc(=O)n(c1)Cc2cc(=O)oc(c23)cc(CC)cc3	20	11.5
Z1323562595	CN(C)C(=O)C1CCCN1C(=O)Nc2ccc(cc2)OCCN3CCN(C)CC3	10.9	19.5
Z28668391	N#Cc1ccc(cc1)C(=O)Nc(cc2)ccc2-c(n3)cn(c34)ccs4	14.9	4.3
Z16825131	c1ccccc1C2=NN(CC2)C(=O)CSc(n3C4CC4)nnc3COc5c(Cl)cccc5	16.7	9.8
Z91710837	Cc1nc(no1)-c2ccc(cc2)OCC(=O)Nc(ccc3)cc3C(=O)Nc(cc4C(F)(F)F)ccc4	15.1	8.1
Z113921749	N#Cc1c(C)c(C)nnc1NCC(CC2)Cn(c23)c(nn3)C(C)C	25.8	10.1
Z25491415	O1COc(c12)ccc(c2)CNC(=O)CSc(n3)n(-c4ccccc4)cc3-c5cc([N+]([O-])=O)ccc5	0.8	9.5
Z52934197	C1CC1NC(=O)CNc2cc(ccc2)S(=O)(=O)N(C)c3ccccc3	5.4	7.7
Z403728182	O1COc(c12)cc(cc2Cl)C(=O)NC(C)c(nn3)n(c34)CCC4	3.9	7.6
Z364198102	c1cccc(c12)oc(c2COC)C(=O)Nc3cc(ccc3)N(C4=O)CCO4	11.8	9.9
Z595913416	C1CCCCC1NC(=O)c2cc(C)c(cc2)NC(=O)NCCCCN3CCOCC3	1.8	6.3
Z91187460	n1cncn1-c(cc2)ccc2C(=O)NCCc(nn3)n(c34)cccc4	23.7	3
Z381656616	Cn(c1)c(=O)[nH]c(=O)c1C(=O)N2CCN(CC2)Cc3csc(n3)-c4ccc(cc4)OC	-4.9	3.3
Z56949551	CCOC(=O)c1c(NC(=O)CCl)cc(s1)-c2ccc(F)cc2	-2.4	3.1

Z785972562	c1ccc(F)cc1CC(=O)Nc(no2)nc2-c3ccccc3	2.2	6.4
Z48851471	c1cccc(Cl)c1Cn(c(C)c2)c(C)c2-c3csc(n3)Nc(c4)ccc(c45)OCCO5	-8.2	10.6
Z220564906	CCc(c1)sc(c12)nc(C)nc2Nc3cc(O)ccc3	1.1	9.3
Z253293494	s1cccc1CN(CC)C(=O)c2cc(n[nH]2)-c(cc3)ccc3C	-3.5	2.4
Z1041113978	c1cc(Cl)cc(c1C)-n(nc2)c(c23)ncnc3Nc4ccc(cc4)Oc5ccc(Cl)cc5	11.4	4.1
Z28655806	s1cccc1\C=C\C(=O)Nc2nnc(s2)SCc3c(Cl)cccc3Cl	2.8	3.4
Z89291848	Cc(c1)ccn(c12)cc(n2)C(=O)Nc(c3)ccc(c34)OCO4	18.7	2.7
Z19514186	CCOc(cc1)ccc1-n2c(SCC(=O)c3cccs3)nnc2-c4c[nH]c(c45)cccc5	-2.8	4.5
Z15656934	CCCC(C)NC(=O)COC(=O)CC(c1ccccc1)NC(=O)c2c(Cl)cccc2	12	12.3
Z381654216	Cc(c1)ccc(c12)[nH]c(c2Cl)C(=O)Nc(c3)ccc(c34)[nH]c(=O)[nH]4	-5.3	4.1
Z225374568	c1ccccc1-c(n2)c(n(c23)cc(C)cc3)NC(=O)CCc4ncc(o4)-c5ccc(Cl)cc5	-7.3	15.2
Z24132420	FC(F)Oc(cc1)ccc1\C=C\C(=O)OCC(=O)NCc2c(Cl)cccc2	6.9	7.7
Z167648830	c1c(F)cc(F)cc1NC(=O)c2cc([N+]([O-])=O)c(cc2)NC3CC3	6.8	12.8
Z226998132	C1COCCN1Cc2ccc(cc2)NC(=O)CSc3ncnc(c34)n(nc4)-c5ccc(F)cc5	8.9	7.6
Z26706965	c1ccccc1CC(C(=O)NC(C)CCC)NC(=O)OCc2ccccc2	-11.7	12.5
Z71577556	c1ccccc1C(=O)c2ccc(cc2)C(=O)Nc(c3O)nccc3	-3.9	14.8
Z25043114	c1ccccc1-n(n2)c(C)c(c2C)NC(=O)COC(=O)c3nc(-c4ccccc4)n(n3)-c5ccccc5	12.6	6
Z88541443	CCNC(=O)C(C)NC(=O)c(c1)c(cc(c12)cccc2)OCc3c(C)noc3C	10.1	6.9

Z365986106	C1CCCCCC1N(C)C(=O)c2n[nH]c(c23)ccc(c3)[N+]([O-])=O	3.5	11.8
Z29325776	Cc1cnc(s1)NC(=O)c2cc(ccc2)S(=O)(=O)N(C)c3cc(Cl)ccc3	-2.4	4.5
Z966181786	FC(F)(F)CCN(CC)C(=O)c1cc(Br)ccc1	20	12.2
Z82156307	c1sccc1C(=O)OCc2csc(n2)Nc3ccc(cc3)OC	-5.8	8.2
Z314617634	c1cc(F)ccc1-n(c(c23)ncnc3O)cc2-c4ccccc4	5.3	9.3
Z44307701	O1COc(c12)cc(cc2OC)/C=C(\C#N)c(n3)[nH]c(c34)cccc4	11.1	3.4
Z372504624	c1[nH]ncc1NC(=O)C2=NN(c3ccccc3)C(C2)c4ccccc4	3.8	8.2
Z15951070	N#Cc1c(Cl)cc(cc1)NC(=O)COC(=O)CC(c2ccccc2)NC(=O)c3ccccc3	2.2	7.7
Z101207250	Cc1cc(O)c(cc1)NC(=O)c2cc(ccc2)NC(=O)OCC	13.1	13.6
Z26860269	CCC(C)c1c(cccc1)NC(=O)c(c2C)oc(c23)ccc(c3)OC	19	9.8
Z56846281	c1ccccc1C[C@@H](NC(=O)c2cccs2)C(=O)OCC(=O)NCc3ccccc3	12.1	6.7
Z255321108	Cc1noc(C(C)C)c1C(=O)Nc2ccc(cc2)Nc3ccc(cc3)OC	-5.2	6.4
Z288788168	c1ccccc1CC(C(=O)NCC2CC2)NC(=O)OCc3ccccc3	-1.1	10.7
Z18159821	s1cccc1CNC(=O)COC(=O)/C=C/c2ccc(cc2)OCC=C	-10	17.3
Z319333156	[nH]1c(=O)oc(c12)ccc(c2)NC(=O)/C=C/c3ccc(o3)C(C4)C4C	-4	20.1
Z1263714226	CCOC(=O)c1ccc(cc1)NC(=O)c(cc2)cc(c23)ncnc3Nc(cc4)ccc4C(=O)OCC	20.4	10.4
Z194461442	Cc1oc(=O)cc(C)c1C(=O)OCc2csc(n2)Nc3ccc(cc3)OC	12	8.3
Z748888318	o1cccc1C(=O)NCCC(=O)Nc(c2)ccc(c23)[nH]c(n3)-c4c(F)ccc(F)c4	3.2	3.2

Z729564206	c1ccccc1\C=C\C(=O)Nc2ccc(cc2)NC(=O)c3c(C)onc3	5.1	8.7
Z50079382	CC1=C/C(CC1)=N\NC(=O)c2c(N)cc(s2)-c3ccc(Cl)cc3	-8.1	6.6
Z16033354	c1ccccc1COC(=O)NC(Cc2c[nH]c(c23)cccc3)C(=O)OCC(=O)Nc4cccc(C)c4C	4.4	11.5
Z56910691	COc(cc1)ccc1CCNC(=O)C(=Cc2ccco2)C(=O)NCCc3ccc(cc3)OC	5.1	7.2
Z993960742	c1ccc(F)cc1CCNC(=O)C(Cc2ccccc2)C(=O)NCCc3cc(F)ccc3	-5.9	7.6
Z57455953	OCCNc1ncnc(c12)n(-c3ccc(F)cc3)cc2-c4ccccc4	2.4	7.4
Z362807152	Cc1cc(Cl)cc(c12)cccn2	11.7	11.3
Z235542858	c1cc(C)nc(c12)cc(Cl)cc2	13.5	8.5
Z295461060	Fc1ccc(C)c(c12)nccc2	-0.2	3.7
Z55928967	Cc1cc(Cl)nc(c12)c(C)ccc2	2.5	3.8
Z57012199	Cc1cc(Cl)nc(c12)cccc2	-5.1	4.5
Z56347058	Cc1cc(Cl)nc(c12)cc(C)cc2	4.6	11.8
Z212170818	Clc1c(C)ccc(c12)cccn2	4.6	6.5
Z1198219341	Clc1c(C)cnc(c12)cccc2	6.7	10.5
Z361878560	c1ccnc(c12)ccc(F)c2	12.6	9.2
Z104485978	c1cccc(c12)nc(Cl)cc2	15.9	8.8
Z431511594	c1c(Br)ccc(c12)nccc2Cl	17.4	5.2
Z415653134	Cc1c[nH]c(c12)cccc2	13.3	17.6

Z1171217428	c1ccc(C)c(c12)[nH]cc2	19.7	7.8
Z1203162600	Cc(c1)[nH]c(c12)ccc(Cl)c2	-5.1	8.6
Z1210889726	c1cc(Br)cc(c12)nccc2Cl	5.8	7.6
Z431511746	c1ccc(Br)c(c12)nccc2Cl	10.1	11.3
Z56926563	c1cc(C)nc(c12)cccc2	20.3	6.1
Z384970524	c1cc(C)cc(c12)[nH]cc2	11.8	7.4
Z57369984	Cc(c1)c(C)cc(c12)n(C)cn2	-20.8	8.6
Z382723676	c1c[nH]c(c12)cc(F)cc2	17	13.8
Z1020823312	Clc1c[nH]c(c12)cccc2	-2	6.1
Z1160902020	c1c[nH]c(c12)ccc(F)c2	16.7	6.6
Z330922562	BrCc1cc(Cl)cc(c12)cccn2	-3.1	9.8
Z330922554	BrCc1c(Cl)ccc(c12)cccn2	2.8	10.8
Z330917140	c1c[nH]c(c12)ccc(c2)-c3ccccc3	-7.6	4.1
Z243277338	n1cccc(c12)cccc2F	-2.7	8.7
Z1203162476	Cc(c1)[nH]c(c12)ccc(F)c2	-3.3	4.8
Z56766547	c1cccn(c12)c(C)c(c2)-c3ccccc3	-5.9	6.9
Z147652344	c1cc(Cl)cc(c12)ncnc2Cl	10.9	6
Z727600806	Clc1ncnc(c12)cccc2F	1.9	3.1

Z119990038	c1cccc(c12)ncnc2Cl	6.7	9.6
Z56924486	Cc1cc(Cl)nc(c12)c(C)cc(C)c2	1.6	0.9
Z384970526	Cc1cccc(c12)[nH]cc2	15	5
Z126917305 9	Brc1ccc(C)c(c12)nccc2	-19.9	7.3
Z53833303	n1cn(C)c(c12)cccc2	14.6	11.4
Z57200682	Cc(c1)[nH]c(c12)cccc2	11.3	3.9
Z119990034	c1c(Cl)ccc(c12)ncnc2Cl	5.4	11.7
Z104472852	c1ccnc(c12)cccc2	5.3	13.6
Z1216814187	CC(C)(C)n(cn1)c(c12)cccc2	13.4	8.4
Z56174838	c1cccc(c12)[nH]c(c2)-c3ccccc3	15.1	16.2
Z57983000	ClCc(c1)c(Cl)nc(c12)cccc2	3.8	10.8
Z316118462	c1c(Br)ccc(c12)ncnc2Cl	7.2	3.1
Z1269215995	c1cc(C)nc(c12)ccc(Br)c2	-2.2	6.9
Z56347310	ClCc(c1)c(Cl)nc(c12)cc(C)cc2	-6.4	10
Z330911506	BrCc1cc(F)cc(c12)cccn2	17.8	17.1
Z57114897	c1ccccc1C(C)n(cn2)c(c23)cccc3	20.5	5
Z900198800	c1ccccc1COc(c2)ccc(c23)nccc3	3.4	13.4
Z223026186	CNC(C)c1ccc(cc1)-n(cn2)c(c23)cccc3	19.3	2.9

Z29546 3532	COc(cc1)ccc1-n(c(c23)ncnc3O)cc2-c4ccccc4	-7.6	15.8
Z55627 443	Cc1cc(C)nc(c12)n(-c3ccccc3)nc2-c(cc4)ccc4C	-8	6.9
Z57456 164	OCCNc1ncnc(c12)n(-c3ccc(cc3)OCC)cc2-c4ccccc4	-2.3	23.6
Z56801 548	Nc1ncnc(c12)n(nc2)-c3ccccc3	-5.9	4.9
Z31460 6428	NNc1ncnc(c12)n(nc2)-c3ccc(Cl)cc3	23.1	19.9
Z68084 2008	O=c1cc(C)[nH]c(c12)n(CCO)nc2-c(c3C)cccc3	-3.7	7.8
Z28578 2396	CN(C)c1ncnc(c12)n(nc2)-c3ccc(F)cc3	5.5	3.8
Z57979 075	CCc1ccc(cc1)-c2nn(c(c23)[nH]c(cc3=O)COC)-c4ccccc4	2.7	5.7
Z57979 355	c1ccccc1-n(c(c23)[nH]c(CC)cc3=O)nc2-c4ccccc4	13.3	6
Z57455 954	OCCCNc1ncnc(c12)n(-c3ccccc3)cc2-c4ccccc4	5.7	7.6
Z57455 872	OCCCNc1ncnc(c12)n(-c3ccc(cc3)OC)cc2-c4ccccc4	-6.7	9
Z22041 6906	CON(C)c1ncnc(c12)n(nc2)-c3ccccc3	-7.2	4.3
Z57455 467	c1ccccc1-c2cn(-c3ccc(F)cc3)c(c24)ncnc4N5CCOCC5	-14.4	14.3
Z22783 2758	Nc1ncnc(c12)n(c(C)c2C)-c3ccc(O)cc3	21.9	7.5
Z90124 734	Nc1ncnc(c12)n(c(C)c2C)-c3ccc(Cl)cc3	6.7	6.6
Z90122 142	Cc1ccc(cc1)-n(c(C)c2C)c(c23)ncnc3N	8	14.7
Z57455 905	c1ccccc1-c2cn(-c3ccc(cc3)OC)c(c24)ncnc4N5CCOCC5	-4.2	13.3
Z56995 257	s1cccc1-c(cc2C(F)(F)F)nc(c23)n(-c4ccccc4)nc3-c(cc5)ccc5C	-3.3	4.7

Z56865447	n1ncn(c1c23)cnc2n(nc3)-c4ccccc4	-11.2	1.9
Z56852588	c1cccc(c12)n(C)c(n2)-c3c(N)n(c(c34)c(=O)n(C)nc4[N+]([O-])=O)C5CCCCC5	-3.2	10.1
Z285749166	n1ncn(c1c23)cnc2n(nc3)-c(c4)ccc(Cl)c4Cl	-10	24.2
Z108567154	n1nnn(c1c23)c(C(F)(F)F)nc2n(nc3)-c4cc(Cl)ccc4	4.5	5.5
Z57455935	COCCNc1ncnc(c12)n(-c3ccc(F)cc3)cc2-c4ccccc4	15.9	10.4
Z680883240	O=C(O)c1cc(C2CC2)nc(c13)n(CCO)nc3-c4cccs4	10.5	10.3
Z57455912	COCCNc1ncnc(c12)n(-c3ccc(cc3)OC)cc2-c4ccccc4	-3.7	15.3
Z56763501	c1ccccc1C(=S)Nc(c2-c3ccccc3)n4nc(n(c4c25)CCCC5)-c6ccc(cc6)OC	-7.9	10.9
Z237576460	N#Cc1cc([N+]([O-])=O)c(cc1)Oc(cc2)ccc2-n(c(C)c3C)c(c34)ncnc4N	-0.4	2
Z211311232	CCNc1ncnc(c12)n(nc2)-c3ccccc3	8.7	5.2
Z220324560	CCNc1ncnc(c12)n(nc2)-c3ccc(F)cc3	4.6	10
Z1218291815	c1cccc(Cl)c1C(O)Cn2cnc(c23)ncnc3NC	7.2	6.8
Z238676528	C1CCN1c2ncnc(c23)n(nc3)-c4ccccc4	2.6	6.6
Z56792085	FC(F)Oc(cc1)ccc1-c2cn3cc(n(c3c24)CCCC4)-c5ccccc5	-3	10.5
Z220404372	C1CC1Nc2ncnc(c23)n(nc3)-c4ccccc4	9.9	5.2
Z220404374	C1CC1Nc2ncnc(c23)n(nc3)-c4ccc(F)cc4	18.4	14.9
Z104111405 8	COc(cc1)ccc1-n(nc2)c(c23)ncnc3NC4CC4	22.7	10.1
Z220403918	OCCN(C)c1ncnc(c12)n(nc2)-c3ccccc3	4.7	8.1

Z921320356	CCCN(C)c1ncnc(c12)n(nc2)-c3ccccc3	7.5	1.4
Z56844776	[O-][N+](=O)c1nn(CC)c(=O)c(c12)n(C3CCCCC3)c(N)c2-c(n4)oc(c45)cccc5	1.6	3.8
Z118438576	C=CCNc1ncnc(c12)n(nc2)-c3ccccc3	10.3	7
Z234904272	O1CCCC1Cn(c(C)c2C)c(c23)ncnc3N	1.8	6.5
Z57456112	CN1CCN(CC1)c2ncnc(c23)n(-c4cc(Cl)ccc4)cc3-c5ccccc5	16.5	10.7
Z57455630	c1ccccc1-c2cn(-c(cc3C)ccc3)c(c24)ncnc4N(CC5)CCN5C	2.2	6.9
Z56881877	c1cc(Cl)ccc1-c2c(C)sc3ncn(c4c23)cnn4	0.7	6.4
Z56792993	C1CCCc(c12)n(-c(cc3C(F)(F)F)ccc3)c(C)c2-c4ccccc4	16.7	3.8
Z56776346	c1ccccc1-c(c2C#N)c(-c3ccccc3)n(c2NC(=O)C)C4CCCCC4	8.8	9.8
Z57455481	COC(OC)CNc1ncnc(c12)n(-c3ccc(F)cc3)cc2-c4ccccc4	21.7	4.3
Z56801378	CCOC(=O)CCn(c(c12)ccc(C)c2)c(-c3ccccc3)c1-c4ccccc4	-5.2	2.9
Z56844779	c1ccccc1CCn(c(c23)c(=O)n(CC)nc3[N+]([O-])=O)c(N)c2-c(n4)sc(c45)cccc5	1	14.7
Z56987685	CC(C)(C)c1ccc(cc1)-c2nn(-c3ccccc3)c(c24)NC(=O)CSC4c5ccc(Cl)cc5	7.9	3.4
Z52590800	c1ccccc1-c2csc(c23)nc(C)nc3Nc(cc4)cc(c4OC)S(=O)(=O)N5CCOCC5	3.5	8.7
Z44121634	n1cccnc1N(CC2)CCN2Cn(c(=S)s3)nc3Nc4ccc(cc4)Oc5ccccc5	-14.3	10.4
Z85937709	c1cc(O)ccc1Nc2nc(CN3CCOCC3)nc(c24)scc4-c5ccccc5	9	9.2
Z55730373	c1cc(Cl)ccc1-n(n2)c(cc2C)NC(=O)c3cccc(n3)N(CC4)CCN4c5ccc(cc5)OC	-4.3	6
Z56854378	CCOC(=O)c1c(-c(cc2)ccc2C)csc1Nc(n3)c(N(CC4)CCN4C)nc(c35)cccc5	-8.5	5.2

Z432419410	c1cc(F)ccc1Nc(cc(n2)C)n(c23)nc(c3)-c4cc(OC)c(cc4)OC	4.1	3.5
Z71175700	COc(c1)ccc(OC)c1NC(=O)C(C)Sc(nc2NC)nc(c23)cccc3	9.7	14
Z31065921	COc1c(O)cc(cc1)Nc2nc(-c3cccnc3)nc(c24)cccc4	1.8	4.1
Z316130872	n1cccnc1N(C2)CCCC2C(=O)Nc(n3)sc(c34)cc(cc4)Oc5ccccc5	8.5	19.8
Z1013695826	c1cc(F)ccc1CNc(nc(c23)nccn3)nc2N4CCN(CC4)c5ccccc5	4.7	2.8
Z85932913	c1ccccc1-c(c2)sc(c23)ncnc3Nc4ccc(cc4)NS(=O)(=O)c(c5)ccc(c56)OCCCO6	0.6	10.2
Z17931156	C1COCCN1c(cc2)ccc2NC(=O)C(C)Sc(ncn3)c(c34)sc(c4)-c5ccc(F)cc5	6.9	9.9
Z85926118	c1ccccc1-n(n2)c(cc2C)Nc(c3Cl)nc(c(Cl)c3)N4CCN(CC4)c5ccc(cc5)OC	-4.1	3.9
Z238653612	c1ccccc1NC(=O)CN(CC2)CCC2Nc3ncnc(c34)cc(Cl)cc4	7.3	22.7
Z56948504	c1ccccc1-n(n2)c(cc2C)Nc(n3)c(N(CC4)CCN4C)nc(c35)cccc5	4.4	7.9
Z332874806	n1cccnc1Nc(ccc2)cc2C(=O)Nc3ccc(nc3)N4CCCC4	-3.1	7.7
Z52588733	CNC(=O)c1ccc(cc1)Nc2ncnc(c23)scc3-c4ccc(cc4)OC	21.8	4.8
Z431951354	COc(cc1)c(OC)cc1-c2c(C)nn(c23)c(cc(n3)C)Nc4ccc(Br)cc4	2.8	6.8
Z127487484	c1ccccc1Nc2cc(ccc2)Nc(nc3)ccc3S(=O)(=O)N4CCOCC4	-10.7	9
Z1041114212	Cc(c1)ccc(c1C)Nc2nc(nc(c23)nccn3)N(CC4)CCN4c5ccccc5	21.1	5.4
Z82240952	CN1CCN(CC1)c(ccc(Cl)c2)c2NC(=O)c3ccc(cc3)-n4cncn4	10.4	9.6
Z435980478	Cc1cc(ccc1)Nc(cc(n2)C)n(c23)nc(c3)-c4cc(OC)c(cc4)OC	-0.6	2
Z431947114	CC(=O)Nc1ccc(cc1)Nc(cc(n2)C)n(c23)ncc3-c4c(OC)cccc4	2.6	22.2

Z1041113928	c1cccc(OC)c1N2CCN(CC2)c(nc(c34)nccn4)nc3Nc5ccccc5	26.2	4.2
Z118515262	COc(cc1)cc(OC)c1Nc(nc2)ccc2-c(n3)[nH]c(c34)cccc4	0.1	3.9
Z445008618	CNc(nc1)ccc1Nc2nc(C)nc(c23)scc3-c4cc(OC)c(cc4)OC	-6.5	4.7
Z31124105	CC(=O)Nc(c1)ccc(OC)c1Nc2ncnc(c23)scc3-c4ccc(cc4)OC	8.8	4.7
Z56765335	c1cc(O)c(OC)cc1/C=N/Nc2nc(C)nc(c23)n(nc3)-c4ccccc4	13	2.3
Z432419392	Cc1ccc(cc1)Nc(cc(n2)C)n(c23)nc(c3)-c4cc(OC)c(cc4)OC	-4.8	9.1
Z57243864	c1cccc(c12)nc(-c3c(O)c(OC)ccc3)nc2N4CCN(CC4)c5ccccc5	-0.9	3.9
Z1041113922	Cc(c1)ccc(c1C)Nc2nc(nc(c23)nccn3)N(CC4)CCN4c5cc(Cl)ccc5	12.1	6.4
Z440824940	c1ccc(Cl)cc1Nc(cc(n2)C)n(c23)nc(c3)-c4cc(OC)c(cc4)OC	9.6	4.2
Z56851745	COc(cc1)cc(OC)c1Nc(nc(c23)ccc(C)c3)nc2-c4ccccc4	-4.9	2.8
Z992054424	c1ccccc1-c2nc(nc(c23)ccc(C)c3)Nc4c(OC)ccc(c4)OC	15.1	11.3
Z114956180	COCCNC(=O)c1cc(ccc1)Nc(nc(c23)ccc(Cl)c3)nc2-c4ccccc4	26.7	7.2
Z16382483	C1COCCN1c(cc2)ccc2NC(=O)C(C)Sc3ncnc(c34)n(nc4)-c5ccccc5	13.6	9.2
Z118523160	n1cnn(c12)c(cc(n2)C)Nc3ccc(cc3)NS(=O)(=O)c(c4)ccc(c45)OCCO5	-0.6	3
Z1385412515	OCC(=O)Nc1cc(ccc1)Nc2ncnc(c23)cc(F)c(F)c3	-6.2	6.2
Z56878976	c1cccc(OC)c1Nc2nc(-c3c(O)cccc3)nc(c24)cccc4	1.2	6.2
Z57030677	COc(cc1)ccc1-c2csc(n2)NC(=O)c3ccc(cc3)Nc4ncnc(c45)cccc5	14.2	8.8
Z20235156	C1COCCN1c(cc2)ccc2NC(=O)C(C)Sc3nc(C)nc(c34)sc(c4)-c5ccccc5	8.9	10.8

Z57243854	Cc1ccc(cc1)Nc2nc(-c3c(O)c(OC)ccc3)nc(c24)cccc4	12	6.6
Z729055996	CNc(nc1)ccc1Nc2nc(-c3cccnc3)nc(c24)cccc4	13.4	5.4
Z19316079	CCN(CC)c1ccc(cc1)NC(=O)CSc2ncnc(c23)cccc3	-2.6	8.9
Z56880410	O=C(O)c1cc(ccc1)NC(=O)CC(C(=O)N2)Sc(c23)ccc(C(F)(F)F)c3	3.6	4.9
Z57393621	c1cccc(c12)NC(=O)C\2=N\NC(=O)c3cc(n[nH]3)-c4cccc(c45)cccc5	22.3	12.6
Z57172312	O=C(O)c1cc(ccc1)CN2C(=O)SC(C2=O)Nc(ccc3)cc3C(=O)O	7.8	11.5
Z166629440	O=C(O)CCC(C(=O)O)NC(=O)c1ccc(cc1)NCc(n2)cnc(c23)nc(N)[nH]c3=O	17.3	5
Z321133248	NC(=O)CSCCC(=O)Nc(s1)ncc1Cc2cc(OC)ccc2	-5.7	8.7
Z56123802	c1cccc(c1C(=O)O)NS(=O)(=O)c(ccc2)cc2NS(=O)(=O)c(cc3)cc(c34)oc(=O)[nH]4	23.2	11.8
Z365691418	[O-][N+](=O)c(c1)ccc(c12)[nH]nc2C(=O)Nc(c3)ccc(F)c3C(=O)OC	-2.2	10
Z18475190	c1cc(F)ccc1C(=O)CCC(=O)OCC(=O)c(c2)ccc(c23)[nH]c(=O)[nH]3	12.2	10.9
Z56801861	Cc(n1)scc1CC(=O)c2c(O)cc(cc2)OCC3C(O)C(O)C(O)C(O3)CO	-3.6	8.1
Z1481182175	C1COCCN1C(=O)COc(cc2)c(OC)cc2CNc(ccc3)cc3-c4ccn[nH]4	22.9	3.3
Z295635132	C1CC(=O)Nc(c12)ccc(c2)OCC(=O)NCCCN3CCN(CC3)c4ncccn4	-3.2	5.3
Z365698030	[O-][N+](=O)c(c1)ccc(c12)[nH]nc2C(=O)NCc3c(cccc3)Cn4ccnc4	-15.1	3.9
Z230163214	Cc1c(S(=O)(=O)N)cc(cc1)NC(=O)c2cc(n[nH]2)-c(cc3)ccc3C	-8	8
Z372731156	c1cccc(Cl)c1C(CC(=O)O)NC(=O)c2cc([nH]n2)C3CC3	0.6	12.2
Z1131229863	c1cccc(COC)c1C(=O)Nc([nH]n2)cc2-c3c(OC)cccc3	14	12.1

Z16202871	c1cccc(c12)NC(=O)C(C)(C)N2C(=O)CN3C(=O)NC(C3=O)Cc4c[nH]c(c45)cccc5	13.7	6.1
Z1514824214	o1cccc1C2NC(=O)NC(=C2C(=O)OCC)CN(C(CO)C3CC3)Cc4ccccc4	19.5	4.1
Z18544371	c1cccc(c12)[nH]nc2C(=O)OCc3cc(=O)oc(c34)cc(O)cc4	8.2	11.6
Z1370992736	o1cccc1C(O)CN(C(=O)c2c(C)[nH]nc2)Cc3ccccc3	2.1	4.6
Z339901228	n1cccnc1Nc(ccc2)cc2C(=O)N(CC)Cc([nH]c3=O)nc(c34)cc(OC)c(c4)OC	-6.1	14.2
Z1335851916	c1ccccc1Cc2cc(c(O)cc2)NS(=O)(=O)c3c[nH]c(c34)nccc4	17.7	11.6
Z805649870	c1ccccc1-c(n[nH]2)cc2CCCN(C)S(=O)(=O)c(c3)ccc(c34)[nH]c(=O)[nH]c4=O	16.4	9.5
Z46301142	c1cccc(c12)NC(=O)CN2C(=O)CN(CC)Cc([nH]c3=O)nc(c34)cccc4	4.1	9.8
Z401995242	c1cc(F)ccc1-c(n[nH]c2)c2CNCC3(CCOCC3)c4ccccc4	-16.8	20.7
Z1403674537	c1ccnc(c12)[nH]cc2CC(=O)NCC(O)COc(cc3C)ccc3	-9.3	5.2
Z827303042	c1cc(F)ccc1CC(C(=O)N)CNC(=O)c2cc([nH]n2)-c3ccc(F)cc3	0.6	11.4
Z1135145466	c1cccc(c12)n(cn2)CC(c3ccc(F)cc3)NCc4c(C)n[nH]c4	2.7	7.6
Z57456069	OCCNc1ncnc(c12)n(-c3ccc(Cl)cc3)cc2-c4ccccc4	-9.3	12.3
Z809088424	O=c1[nH]c(=O)[nH]c(c12)ncc(c2)C(=O)N(C)CCCCCc3cc(n[nH]3)-c4ccc(F)cc4	22.9	8.2
Z927363860	FC(F)(F)c1cc(c(=O)[nH]c1)NC(=O)CC2C(=O)Nc(c23)cccc3	18.6	13.5
Z758039314	n1cccnc1NCCC(=O)NCc2cc(ccc2)NC(=O)c3ccccn3	12.4	8.1
Z16151137	N1CCC/C1=N/S(=O)(=O)c(ccc2)cc2NC(=O)COC(=O)c3c(cccc3)NCc4ccco4	10	10.3
Z239342080	Cn1ccnc1C(c2ccccc2)NC(=O)c3cc(n[nH]3)-c4ccc(Br)cc4	13.4	11.5

Z992080264	c1cnc(O)cc1CNC(=O)CCc2ncc(o2)-c3ccccc3	-12.7	7.6
Z68340575	c1cccc(c12)cc(O)c(c2)C(=O)NNC(=O)c3c(O)nccc3	17.3	8.3
Z56772943	c1cccc(c1C(=O)O)Nc(cc2C(=O)O)nc(n2)Nc(c3C(=O)O)cccc3	3.6	7.3
Z653213378	CC(C)c([nH]n1)cc1C(=O)NCc2c(nccc2)OCc3ccccc3	-6.3	10.5
Z225416380	Cc1ccc(cc1)-c(n[nH]2)cc2C(=O)NCc3c(cccc3)Cn4cccn4	-5.7	2
Z368234966	c1ccc(NC(=O)N)cc1NC(=O)c2cc([nH]n2)-c3cc(Cl)ccc3	6.8	3.3
Z56773235	O=C(O)c1cc(ccc1)Nc2ccnc(n2)Nc(ccc3)cc3C(=O)O	-2.6	6.5
Z806906560	c1ccnc(O)c1C(=O)NC2CCCOc(c23)ccc(F)c3	19.8	3.4
Z1242032187	c1n[nH]c(c12)CCN(C2)C(=O)c3c(O)ccc(c3)OCc4ccccc4	20.7	7.9
Z211321404	Cc1ccc(cc1)-c(n[nH]c2)c2CNCc3cc(OC)c(cc3)OC	12.1	11.1
Z89761882	c1ccccc1C(=O)CCC(=O)OCc2nc(nc(n2)N)Nc3ccccc3	19.6	11
Z57101386	O=C(O)c1ccc(cc1)Nc2ncnc(c23)n(nc3)Cc4ccccc4	14	6.8
Z1444463148	c1ccccc1C(C)(C(=O)O)NC(=O)Cc2n[nH]c(c23)cccc3	26.3	2.9
Z635171180	c1ccc(OC)cc1-c(n[nH]c2)c2CNCc3c(cccc3)Cn4cncn4	-3.7	9.2
Z56865652	c1cc(Cl)ccc1C(=O)C2=C(O)C(=O)N(CCOC)C2c3cc(OC)c(O)cc3	-1.4	3.2
Z408136442	C1CCCC1C(c2cccs2)NC(=O)c3n[nH]c(c34)ccc(c4)[N+]([O-])=O	3.5	9.4
Z237625012	c1cccc(c12)NC(=O)CN2C(=O)CCC(C3)C(=O)Nc(c34)cccc4	17.8	13.6
Z365094382	CN(C)Cc1cc(ccc1)CN/C=C\2C(=O)NC(=O)c(c23)cccc3	20.8	12.5

Z1019561132	CC1CN(CC(O1)C)C(=O)C(=O)Nc(c(=O)[nH]c2)cc2C(F)(F)F	11.8	10.5
Z318699644	CCCc(n[nH]1)cc1C(=O)Nc(c2)ccc(Cl)c2C(=O)N	18.8	12.2
Z647306402	c1cccc(Cl)c1C(CC(=O)O)NC(=O)c2c(=O)[nH]c(C)cc2	0.4	3.1
Z237057236	COc(cc1)ccc1-c(n[nH]c2)c2C(=O)NNc(n(c3=O)CC)nc(c34)cccc4	10.7	10.7
Z507535496	FC(F)(F)c1cc(c(=O)[nH]c1)NC(=O)CCc2c(C)nc[nH]c2=O	22.8	10.7
Z56774535	O=C(O)c1cc(ccc1)NC(=O)CC(C(=O)N2)Nc(c23)cc(C)c(C)c3	14.7	7.2
Z217469786	CCOC(=O)C(C(=O)OCC)NC(=O)CC(C(=O)N1)Sc(c12)ccc(Cl)c2	-2.4	15
Z653214180	c1cc(C)[nH]c(=O)c1C(=O)NCc2c(nccc2)OCc3ccccc3	2.1	8.9
Z1173966611	CCC(C)C(C(=O)OC)NC(=O)c(c1)cnc(c12)n[nH]c2C	10.3	2.6
Z928388106	O=C(O)c1cc(ccc1)NC(=O)c2cc([nH]n2)C3CC3	-15.5	11.3
Z24427216	COc(c1)ccc(O)c1C(=O)OCC(=C2C(=O)OCC)NC(=O)NC2CC	8.1	5
Z1128987919	CCCc(n[nH]1)cc1C(=O)NC(c2nc(C)[nH]n2)c3ccccc3	13.5	8.4
Z15517071	CC(=O)c1c(C)c([nH]c1C)C(=O)OCC(=O)Nc(cccc2)c2C(=O)Nc3ccccc3	8.1	12.2
Z1039510912	FC(F)(F)c1cc(c(=O)[nH]c1)NC(=O)c(c2)c(O)cc(c23)OCO3	25.3	4.4
Z15517598	CC(=O)c1c(C)c([nH]c1C)C(=O)OCC(=O)NC(C)c2ccccc2	9.6	18.8
Z777307318	CC(C)c1nc([nH]n1)-c2c(cccc2)NC(=O)c3cc(ccc3)OCc(n4)cn(c45)cccc5	-19.8	9.9
Z645179942	CC1CN(CC(O1)C)c(nc2)ccc2CNc(n(c34)ncn3)cc(n4)-c5ccccc5	18.5	8.6
Z852105782	N1CCN(C1=O)c(cc2)ccc2NC(=O)c3cc(C4CC4)nc(c35)n(nc5C)-c6ccccn6	-11.7	7.8

Z90623596	c1cccn(c12)c(nn2)CNCc3c(-c4c(OC)ccc(c4)OC)nn(c3)-c5ccccc5	-9.2	8.5
Z981162044	c1cccc(c12)c(=O)n(C(C)C)nc2C(=O)Nc3ccc(nc3)Nc4ccccc4	-7.4	7.3
Z816588536	Cc1c[nH]c(=O)n1-c(cc2)ccc2C(=O)Nc3c(nccc3)Oc4cccc(C)c4C	11.1	13.1
Z1424434795	c1ccccc1-c(nc(c23)n(C(C)C)nc2)cc3C(=O)Nc(cc4)ccc4Cc5nnn[nH]5	5.4	4.6
Z225084586	CC(C)n(nc1)c(c12)nc(-c(c3C)cccc3)cc2C(=O)Nc(ccc4)cc4-c5nnn[nH]5	-0.3	11.7
Z1613974092	c1cccc(c12)nc(-c3ccncc3)cc2C(=O)Nc(c4)ccc(c45)[nH]c(CC)cc5=O	-10.8	6.8
Z916530322	c1ccccc1-c(nc(c23)n(C(C)C)nc2)cc3C(=O)NCc4ccc(cc4)-c5nnn[nH]5	-11.9	6
Z786038522	c1nccn1CCC(=O)Nc(ccc2)cc2C(=O)Nc(s3)nc(c3C)-c4ccc(F)cc4	-17.4	5.9
Z237087286	c1ccnc(c12)c(ccc2)NC(=O)c3ccc(cc3)OCc(n4)cn(c45)cccc5C	-3.6	4.9
Z460286118	c1cc(F)cc(Br)c1Oc(nccc2)c2C(=O)Nc(c3)cnc(c34)n(C)nc4C	14.1	6.3
Z645152226	COc(cc1)c(O)cc1CNc2ncnc(c23)scc3-c4ccc(cc4)OC	-5.5	7
Z261772600	c1n[nH]c(c12)ccc(c2)NC(=O)c3ccc(cc3)-n(c4=O)c(C)nc(c45)cccc5	11.7	11.1
Z240692216	CN(C)c(cc1)ccc1C(=O)Nc(s2)nc(-c3ccccc3)c2-c4nccn4C	4.7	12.8
Z55701717	c1n[nH]c(c12)cc(cc2)Nc(n3)c(nc(c34)cccc4)NS(=O)(=O)c(c5F)cc(F)cc5	1.7	8.2
Z786028994	c1nccn1CCC(=O)Nc(ccc2)cc2C(=O)Nc(c3)ccc4n(CC)c(c5c34)cccc5	-7.3	10.2
Z856407970	n1ccccc1-n(c(C)c2)c(C)c2C(=O)Nc(c3C)ccc(c3)Oc4ncccn4	-8.5	4.9
Z940773446	c1cc(Br)ccc1C(O)C(C)Nc2nc(-c3ccncc3)nc(c24)cccc4	2.4	5.3
Z31225677	c1cccc(c12)nc(-c3cccnc3)nc2Nc4ccc(cc4)N5CCOCC5	8.5	6

Z1459140404	c1cc(F)ccc1-c(nc(c23)cccc3)cc2C(=O)Nc4ccnn4-c(nn5)ccc5C	-6.8	5
Z1157725078	COc(cc1)ccc1CNc(nc(c2c34)cc(OC)c(c2)OC)n4nc(n3)-c5cccnc5	11.3	8.7
Z1041113992	Cc1c(C)ccc(c1)-n(nc2)c(c23)ncnc3Nc4ccc(cc4)Oc5ccccc5	19.5	12
Z914110990	CN(C)c1ccc(nc1)NC(=O)c2c(C)n(c(C)c2)-c(c3)ccc(c34)OCCO4	-8.7	3.6
Z1157725081	c1ccccc1CN(C)CCCNc(nc(c2c34)cc(OC)c(c2)OC)n4nc(n3)-c5cccnc5	4.3	9.1
Z1041114046	Cc1c(Cl)cc(cc1)-n(nc2)c(c23)ncnc3Nc4ccc(cc4)Nc5ccccc5	13.9	5.3
Z31244267	COc(nc1)ccc1Nc2nc(-c3cccnc3)nc(c24)cccc4	13.2	7.4
Z1165302444	c1cccc(OC)c1CNc(nc(c2c34)cc(OC)c(c2)OC)n4nc(n3)-c5cccnc5	-16.4	7.9
Z1013695848	c1ccccc1CNc2nc(nc(c23)nccn3)Nc(cc4OC)cc(OC)c4OC	8.1	13
Z1544663259	c1cccc(c12)[nH]c(n2)/C=N/Nc(nc(c34)cc(C)cc4)nc3-c5ccccc5	-4.9	12.6
Z1541746259	Cc1nncn1-c(ccc2)cc2NC(=O)c3cc(OC)c(c(c3)OC)OCc4ccccc4	-16.3	10.8
Z318116494	C1COCCN1c(nccc2)c2C(=O)Nc(n3)scc3-c(cc4C(F)(F)F)ccc4	-24.9	7.2
Z952594020	C1CCCC1NC(=O)Cn(c2)ncc2NC(=O)c3cc(-c4ccccc4)c(s3)N5CCOCC5	8.2	8.3
Z1513492613	n1c[nH]c(c12)ccc(c2)C(=O)N3CCN(CC3)c4nc(-c5cccnc5)nc(c46)cccc6	-14.7	8.5
Z1528739220	n1ncn(C)c1-c2cc(cnc2)NCc3c(-c4ccc(F)cc4)nn(c3)-c5ccccc5	6.2	15.3
Z49539457	Cc1cc(C)nc(n1)N\N=C\c2c(-c(c3)ccc(c34)OCCO4)nn(c2)-c5ccccc5	9.7	4.5
Z1410912518	CCOC(=O)c1c(nccc1)NC(=O)c2cc(-c3ccccc3)nc(c24)n(C)nc4C	-1.5	6
Z1041114040	Cc1c(Cl)cc(cc1)-n(nc2)c(c23)ncnc3Nc4ccc(cc4)Oc5ccccc5	13.8	11.8

Z1041113994	Cc1c(C)ccc(c1)-n(nc2)c(c23)ncnc3Nc4ccc(cc4)Oc5ccc(Cl)cc5	11	8
Z1157725082	Cc1cc(ccc1)N(CC)CCCNc(nc(c2c34)cc(OC)c(c2)OC)n4nc(n3)-c5cccnc5	-0.8	12.3
Z750137620	c1cccc(c12)n(C)c(c2Cl)C(=O)Nc3ccc(nc3)Oc(cc4)c(OC)cc4C	-2.5	12.3
Z1436083684	Cc1cnc(nc1)NC(=O)Cc2c(C)n(c(c23)ccc(c3)OC)C(=O)c4ccc(Cl)cc4	-2.6	6.9
Z100355784	c1cc(N)ccc1C(\C)=N/Nc2ncnc(c23)scc3-c4ccc(F)cc4	17.8	9.4
Z1625059400	c1nc(C)ccc1\C=C\C(=O)Nc(c2C)c(ccc2)-c(n3C(C)C)nc(c34)ccc(F)c4	-5.3	12.4
Z969015972	Cc(n1)n(C)c(c12)ccc(c2)C(=O)Nc(c3C)cccc3NC(=O)c4ccccc4	-0.3	9.3
Z86317498	Nc1[nH]nc(c1C#N)CCCNc2nc(-c3cccnc3)nc(c24)cccc4	6.5	6.3
Z16002408	CC(=O)c1cc(ccc1)NC(=O)CSc(n2-c3c(F)cccc3)nnc2-c4c(Cl)cccc4	1.7	5.1
Z785983240	c1cccc(c2=O)c1n(c(c23)cccc3)CC(=O)Nc4c(ccc(F)c4)NC(=O)c5ccccn5	-14	3.7
Z217126442	c1ccccc1Nc(nc(n2)N)nc2C(\C#N)=C/c3c(-c4cccnc4)nn(c3)Cc5ccccc5	9.1	3.7
Z927306062	O=c1[nH]cnc(c12)ccc(c2)NC(=O)c3c(nccc3)SCc4ccc(F)cc4	-13.9	10.9
Z90537512	c1cc(O)ccc1C(O)C(C)NCc2ccc(o2)-c(n3)sc(c34)cccc4	10.4	7.1
Z31372202	c1cccc(Cl)c1COCC(O)CNCCNCC(O)COCc2c(Cl)cccc2	13.4	8.7
Z124831322	c1ccccc1CNC(=O)NC(=O)C(C)NCC(c2ccccc2)c3c[nH]c(c34)cccc4	-0.9	3.9
Z57110353	c1ccccc1CNCC(O)COc2ccc(cc2)OCC(O)CNCc3ccccc3	8.3	9.9
Z1373944652	Cc1c(Cl)cc(cc1)NC(=O)c2c(nccc2)Oc(c3C(=O)N)cccc3	4.6	5.2
Z739801546	Fc1c(Cl)cc(cc1)NC(=O)CNc2c(cccc2)N(CC3)CCC3O	15.4	7.7

Z106085620	c1cccc(c12)nc(n(c2=O)CC(=O)N)CNC(C)c(c3)ccc(c34)cccc4	1	6.8
Z606268776	c1cccc(c12)[nH]c(n2)C(Cc3ccc(O)cc3)NC(=O)COc(c4)ccc(c45)CCC5	-27	5.8
Z126204244	Fc1c(Cl)cc(cc1)NC(=O)C(C)N(CC2)CCC2C(=O)Nc3c(O)cccc3	1	19.9
Z1431645389	Fc1c(Cl)cc(cc1)NC(=O)CNc2c(ccc(Cl)c2)N(CC3)CCC3CO	7.8	11.5
Z211321200	c1cccc(c12)[nH]cc2CCNCc3c(Br)cc(cc3)OCCO	-12.4	19
Z25766533	Fc1c(Cl)cc(cc1)NC(=O)Cn(c(c23)cccc2)c(n3)NCC(C)O	7.5	2.8
Z44584637	O1COc(c12)ccc(c2)CNC(=O)NCC3CC(CCC3)CNC(=O)NCc(c4)ccc(c45)OCO5	-0.7	13
Z126201948	c1cc(F)ccc1NC(=O)C(C)N(CC2)CCC2C(=O)Nc3c(O)cccc3	2.9	6.1
Z940669346	c1ccc(Cl)cc1NC(=O)NC(C)C(O)c2ccc(Br)cc2	-1.7	7.4
Z295870622	c1ccc(F)cc1NC(=O)C(C)Nc(ccc2)cc2NC(=O)Nc3ccccc3	-2.5	8.7
Z425151240	c1ccccc1-n2c(=S)[nH]cc2C(=O)NC(C)c3ccc(cc3)NC(=O)Nc4ccccc4	8.6	12.1
Z739801234	c1c(Cl)ccc(F)c1NC(=O)CNc2c(cccc2)N(CC3)CCC3O	8.2	7.5
Z44571697	c1ccccc1C(=O)Nc2c(OC)cc(c(c2)OC)NC(=O)Nc3cc(Cl)ccc3	2.7	7.1
Z356182964	n1c[nH]c(c12)ccc(c2)C(c3ccccc3)NC(=O)NCC(O4)COc(c45)cccc5	5.4	5
Z648058210	c1cc(F)ccc1NC(=O)NC2CCN(CC2)CC(O)c(c3)ccc(c34)OCO4	2.1	7.8
Z30701269	c1cccc(c12)[nH]c(n2)C(C(C)C)NC(=O)C(C)NC(=O)Nc3ccccc3	9.2	6.5
Z86105202	Cc1cc(ccc1)NC(=O)CCCSc(nc2N)nc3sc(c4c23)CCCC4	5.8	4.9
Z798237180	s1cccc1C(CC)NC(=O)NCc2cc(ccc2)NC(=O)Nc(cc3C)ccc3	19.7	13.9

Z29395265	c1cccc(c12)[nH]c(n2)C(CCSC)NC(=O)CCCc3c[nH]c(c34)cccc4	14.6	9.2
Z381527500	Clc1c(Cl)ccc(c1)NC(=O)NCC(O)c2c(OC)ccc(c2)OC	-0.9	1.3
Z254586188	c1cccc(OC(F)F)c1C(=O)Nc2ccc(cc2)NC(=O)Nc3ccccc3	5.3	14.1
Z126201836	Cc1cc(ccc1)NC(=O)C(C)N(CC2)CCC2C(=O)Nc3c(O)cccc3	18.5	12.2
Z44589929	CN(C)c1ccc(cc1)NC(=O)NCC2CC(CCC2)CNC(=O)Nc(cc3)ccc3N(C)C	-7.6	8.3
Z1451184493	CC(C1)C1C(=O)Nc(cc2)ccc2C(=O)NC3CCCN(C3)Cc4c(O)ccc(Cl)c4	-2.7	9.4
Z1168333976	c1ccccc1NC(=O)Nc2cccc(c23)CN(CC3)CC(O)Cn4cccn4	14.8	13.3
Z254578176	COc(cc1)ccc1CC(=O)Nc2ccc(cc2)NC(=O)Nc3ccccc3	3.4	19.4
Z73272361	C1COCCN1c(ccc(Cl)c2)c2NC(=O)c3ccc(cc3)NC(=O)Nc4ccccc4	8.4	4.3
Z1323575136	C1CC1NC(=O)C2CCCN2C(=O)Nc(c3C)cc(cc3)NC(=O)Nc4ccccc4	23.6	17.7
Z744240036	Cc1cc(ccc1)OCCNC(=O)NC(Cc2ccc(O)cc2)c(n3)[nH]c(c34)cccc4	4.9	6.1
Z240682516	c1cccc(c12)n(C)cc2C(=O)Nc3cc(ccc3)NC(=O)Nc4ccccc4	-8.2	11.4
Z169628894	c1ccccc1CNC(=O)NCC(=O)NCC(c2c(Cl)cccc2)c3c[nH]c(c34)cccc4	21.3	17.7
Z287158648	O1CCCC1C(=O)Nc(ccc2)cc2C(=O)NCC(c3cccs3)c4c[nH]c(c45)cccc5	3.1	14.8
Z647164240	FC(F)(F)c1ccc(cc1)NC(=O)CN(c(c23)cccc2)C(C)CC3C(=O)N	-3.2	8.7
Z236585140	o1cccc1C(=O)Nc(cc2)ccc2CNC(=O)NC3CCN(C(C)C)CC3	6.8	13.6
Z433574908	c1occc1C(=O)Nc(c2)ccc(c2C(F)(F)F)NC(=O)NC3CCCC3	-7.8	5.1
Z240995548	COc(cc1)ccc1NC(=O)CC(C(=O)Nc(c23)cccc2)N3CC(O)c4ccc(F)cc4	-6.7	25.3

Z91116397	c1cccc(SCC(=O)N)c1NC(=O)c2c(-c3cccs3)nn(c2)Cc4ccccc4	-3.9	12.5
Z31380858	c1ccccc1COCC(O)CNCc2ccc(cc2)CNCC(O)COCc3ccccc3	9.7	3.5
Z281074182	CCCNC(=O)C1CCN(CC1)C(=O)NCC(c2cccs2)c3c[nH]c(c34)cccc4	1.8	20
Z126204560	c1ccccc1-c2c(cccc2)NC(=O)C(C)N(CC3)CCC3C(=O)Nc4c(O)cccc4	7.3	9.1
Z45261520	Fc1c(F)c(F)ccc1NC(=O)C(C)Nc2c(OC)ccc(c2)NC(=O)C	-11.9	9.1
Z381529764	c1cccc(c12)cccc2NC(=O)NCC(CN(C)C)C(O)c3ccccc3	1.7	4.4
Z355318320	c1ccccc1C(C(=O)N)Nc(cc2)cc(Cl)c2OCc3cc(F)ccc3	8.2	9.5
Z44580929	O1COc(c12)ccc(c2)NC(=O)NCC3CC(CCC3)CNC(=O)Nc(c4)ccc(c45)OCO5	-4.3	9.1
Z287960806	O1CCCC1C(=O)Nc(ccc2)cc2C(=O)Nc3ccc(cc3)Nc4ccccc4	9.8	24.1
Z254579510	O1CCOc(c12)ccc(c2)C(=O)Nc3ccc(cc3)NC(=O)Nc4ccccc4	22	15.1
Z572584132	c1cccc(c12)[nH]c(n2)C(C(C)CC)NC(=O)CSCC(=O)Nc(cc3)ccc3C	-1.4	7.6
Z991868260	CCn1cc(Br)cc1C(=O)Nc2ccc(cc2)NC(=O)Nc3ccccc3	1.1	10.2
Z254577618	Clc1cccc(F)c1C(=O)Nc2ccc(cc2)NC(=O)Nc3ccccc3	-15.6	4.8
Z275767706	o1cccc1CNCC(O)COc2ccc(cc2)OCC(O)CNCc3ccco3	-8.1	10
Z235444660	c1cc(Cl)cc(Cl)c1C(=O)Nc2ccc(cc2)NC(=O)Nc3ccccc3	1.8	8
Z232521908	O1COc(c12)ccc(c2)NC(=O)C(C)Nc(cc3)cc(Cl)c3NC(=O)C	12.5	9
Z254580030	c1ccc(F)cc1OCC(=O)Nc2ccc(cc2)NC(=O)Nc3ccccc3	11.4	8.4
Z235444696	COc(c1)cc(OC)cc1C(=O)Nc2ccc(cc2)NC(=O)Nc3ccccc3	-4.5	8.7

Z1021244206	c1cc(F)c(F)cc1C(O)CNC(=O)Nc(c2)ccc(c23)n(cc3)CC(C)C	13.2	14
Z254578408	c1cccc(OCC)c1C(=O)Nc2ccc(cc2)NC(=O)Nc3ccccc3	12.3	8.3
Z56821290	c1cc(Br)ccc1NC(=N)Nc(n2)nc(cc2C(F)(F)F)-c3cccs3	9.1	12.4
Z254585582	C/C=C/c1cc(OC)c(cc1)OCC(=O)Nc2ccc(cc2)NC(=O)Nc3ccccc3	-3.8	18.9
Z650998198	O1CCCC1CCC(=O)NC(C)c2ccc(cc2)NC(=O)Nc3ccccc3	2.3	4.6
Z237465434	c1ccccc1SCCCC(=O)Nc2c(cccc2)NC(=O)Nc3ccccc3	18.2	5.6
Z44555139	CCNC(=O)NC(=O)C(C)NCC(c1ccccc1)c2c[nH]c(c23)cccc3	-0.4	7.3
Z44462183	CC(=O)NCCNC(c1ccccc1)C(=O)Nc(c2)ccc(Cl)c2C(F)(F)F	12.3	4.9
Z402485978	CCC(=O)Nc(cc1)ccc1C(C)NC(=O)NCc(c2)ccc(c23)OCCO3	13.5	12.4
Z316142862	c1cccc(c12)[nH]c(n2)C(C(C)C)NC(=O)CCCNC(=O)c3ccsc3	0.8	1.2
Z44480563	CC(=O)Nc(cc1)ccc1NC(=O)C(C)NC(C)c2cccc(c23)cccc3	15.8	5
Z223473910	FC(F)(F)c(c1)ccc(c12)SC(C(=O)N2)CC(=O)NC(Cc3ccccc3)c(n4)[nH]c(c45)cccc5	7	5.3
Z29394570	c1cccc(c12)[nH]c(n2)C(CCSC)NC(=O)CSCC(=O)Nc3c(F)cccc3	10.3	4.8
Z356645216	c1cccc(F)c1C(NC(=O)N)CC(=O)NC(c2cc(C)c(C)cc2)c3ccccc3	3.5	3.3
Z221578000	c1cccc(c12)[nH]c(n2)C(CCSC)NC(=O)CNC(=O)NCc3ccccc3	12	5.6
Z98335631	O1CCCOc(c2)c1cc(nc3N=C(N)N4)c2n3C45CCC(CC5)c6ccccc6	10.5	6.8
Z29394621	c1cccc(c12)[nH]c(n2)C(CCSC)NC(=O)CSC(C)c(n3)[nH]c(c34)cccc4	-10.5	13.4
Z339850870	c1cccc(c12)nc(n(c2=O)CC)NNC(=O)Cc3c(C)[nH]c(c34)ccc(F)c4	-2.3	8.2

Z216438238	[nH]1c(=O)[nH]c(c12)ccc(c2)NC(=O)CCc(c3C)c(C)nc4n3nc(c45)cccc5	2.4	6.6
Z822251060	CC(=O)N(CC1)CCC1C(=O)Nc([nH]2)nnc2-c3cccc(c34)cccc4	14.1	10.5
Z86980111	c1cc(C)c([N+]([O-])=O)cc1C(=O)Nc([nH]2)nnc2-c3ccccc3	4.1	19.2
Z119762404	c1cc(C(F)(F)F)ccc1C(=O)Nc([nH]2)nnc2-c(cc3)ccc3C	20.7	14.1
Z653346990	O1CCCC1C(=O)Nc(ccc2)cc2C(=O)Nc([nH]3)nnc3-c4ccccn4	1.9	3
Z107733062	c1cc(F)ccc1C(=O)Nc(n[nH]2)nc2-c3cc(OC)c(cc3)OC	9.4	8.1
Z994388488	COc(cc1)ccc1N2CC(CC2=O)C(=O)Nc([nH]n3)nc3-c4ccc(Cl)cc4	11.7	8.2
Z992122880	c1ccccc1-c(n[nH]2)nc2CNC(=O)c3ccc(o3)-c(n4)sc(c45)cccc5	8	3.8
Z755516366	c1cccc(F)c1CN(C)C(=O)C(=O)Nc(c2)ccc(c23)NC(=O)CC3	-9.5	5
Z119398974	c1cccc(c12)nc(cc2)C(=O)Nc([nH]n3)nc3-c4ccccc4	17.6	7
Z1024461452	CC(C)C(=O)N(CC1)CCC1Nc2cncc(n2)-n(n3)c(C)cc3C	-9.9	7.8
Z229336870	CCN(CC)Cc1ccc(cc1)CNC(=O)c2ccc(cc2)-c3nnc(o3)-c4ccccc4	0.7	4.1
Z1024462168	Cc1cc(C)n(n1)-c2cncc(n2)N(CC3)CCC3c4n(C)cnn4	15.7	6.9
Z1359494878	c1cc(O)ccc1CCNc(nc2)ccc2-c([nH]c3=O)nc(C)c3C	2.9	6.4
Z119705550	c1cnccc1C(=O)Nc([nH]2)nnc2-c3ccc(F)cc3	-6	12.3
Z1024476606	Cc1cc(C)n(n1)-c2cncc(n2)N(CC3)CCC3N(C4=O)CCN4	-9.7	7.5
Z28003639	S1CCSC1c(cc2)ccc2C(=O)N(C(C)C)Cc3nnc(o3)-c4ccccc4	-0.8	11
Z116818873 9	c1cccc(F)c1CC(C)Nc(nc2)ccc2-c(n3)[nH]c(=O)cc3CC	-0.6	6.7

Z108548800	C1CCCC1C(=O)Nc([nH]n2)nc2-c3ccc(F)cc3	5.9	9.6
Z1262402929	CCOC1CC(C12CCC2)Nc(nc3)ccc3-c(n4)[nH]c(=O)cc4C	1.7	7.2
Z942807650	C1CC(=O)N(C)N=C1C(=O)Nc([nH]2)nnc2-c(s3)ccc3C	3.8	11.9
Z1004045752	c1ccccc1C2CCCN2C(=O)Nc(n3)nncc3-c4ccccc4	-4.3	4.5
Z1168190911	c1ccc(C)c(c1C)OCC(O)CNc(nc2)ccc2-c(n3)[nH]c(=O)cc3CC	4.2	10
Z826946386	Cc1cc(C)n(n1)-c(n2)cncc2NC3CCCCCCC3	-14.3	6.9
Z1029950632	C1CCCCCN1C(=O)Cn(n2)cnc2NC(=O)c3ccco3	5	16.4
Z1272165534	c1n[nH]c(C)c1CCCNc(nc2)ccc2-c(n3)[nH]c(=O)cc3C	0.9	12.4
Z1401275534	Cc1cc(C)n(n1)-c2cncc(n2)N(C3)CCC34CCOC4	3.7	4.6
Z218756846	CC(C)(C)c1ccc(cc1)-c2nnc([nH]2)NC(=O)c3ccc(cc3)N(C4=O)CCN4	14.3	8.4
Z119764216	Cc1ccc(cc1)-c2nnc([nH]2)NC(=O)c3ccc(cc3)OCC4CCCO4	6.2	14.5
Z119632178	O1CCOc(c12)ccc(c2)C(=O)Nc(n[nH]3)nc3-c4ccc(cc4)OC	14.3	3.5
Z108548594	c1cc(F)ccc1C(=O)Nc([nH]n2)nc2-c3ccc(F)cc3	19.8	7.1
Z648160558	c1ccccc1C(C)CC(=O)Nc([nH]2)nnc2-c3c(OC)cccc3	6.2	8.7
Z653348450	CC(C1)C1C(=O)Nc(ccc2)cc2C(=O)Nc([nH]3)nnc3-c4ccccn4	8.1	7.3
Z119400894	c1ccccc1-c2nc([nH]n2)NC(=O)C(CC3=O)CN3c4cccc(C)c4C	5.4	10.5
Z1024461324	CC(C)(C)C(=O)N1CCN(CC1)c(n2)cncc2-n(n3)c(C)cc3C	22.2	20.6
Z826949860	Cc1cc(C)n(n1)-c2cncc(n2)N(CC3)CCC34OCCO4	-8.7	13.3

Z1310760450	C1CCC(C)C(C1C)NC(=O)Nc(n2)nncc2-c3ccccc3	13.6	11.1
Z119631624	c1cc(C(F)(F)F)ccc1C(=O)Nc([nH]2)nnc2-c3ccc(cc3)OC	-26.1	1.6
Z916731876	n1n[nH]c(c12)ccc(c2)C(=O)Nc([nH]3)nnc3-c4c(OC)cc(cc4)OC	24.4	11.5
Z960496910	FC(F)(F)c1nnc([nH]1)NC(=O)c2cc(ccc2)-n3cnnn3	0.7	23.4
Z119631614	c1cc([N+]([O-])=O)ccc1C(=O)Nc([nH]2)nnc2-c3ccc(cc3)OC	2.8	18.5
Z119762342	c1cccc(Br)c1C(=O)Nc(n[nH]2)nc2-c(cc3)ccc3C	1.6	5.1
Z86979651	c1cc(C(F)(F)F)ccc1C(=O)Nc([nH]n2)nc2-c3ccccc3	19.5	13.9
Z119631558	c1ccc([N+]([O-])=O)cc1C(=O)Nc([nH]2)nnc2-c3ccc(cc3)OC	-14.4	10.9
Z119762370	c1cc(Cl)ccc1C(=O)Nc([nH]2)nnc2-c(cc3)ccc3C	10.2	2.3
Z86979625	c1ccc([N+]([O-])=O)cc1C(=O)Nc([nH]n2)nc2-c3ccccc3	-4	6.6
Z231957836	C1CC1N(CC2)CCC2NC(=O)c3ccc(cc3)-c4nnc(o4)-c(cc5)ccc5C	3	6.4
Z1276455741	CNC(=O)c1cc(ccc1)CSCc2nnc(o2)-c3ccccc3	-10.5	3.9
Z1134143861	CCc1cc(=O)[nH]c(n1)-c2ccc(nc2)NCc3cc(F)cc(F)c3	25	8.1
Z942809898	O=c1[nH]c(=O)[nH]c(C)c1CCC(=O)Nc([nH]2)nnc2-c(s3)ccc3C	24.8	6.3
Z1168194023	CCc1cc(=O)[nH]c(n1)-c2ccc(nc2)NCc3ccc(cc3)-n4ccnc4	26	4.7
Z212461372	Cc1n(C)nc(C)c1CCC(=O)Nc(n2)scc2-c3ccc(cc3)OC	2.6	15.1
Z1134134577	CCc1cc(=O)[nH]c(n1)-c2ccc(nc2)NCc3ccc(F)cc3	0.6	16.6
Z26710003	c1cccc(OC)c1CNC(=O)c2ccc(o2)-c(n3)sc(c34)cccc4	11.3	5.1

Z1172237967	CCc1cc(=O)[nH]c(n1)-c2ccc(nc2)NCc3ccco3	8.1	14.4
Z119636060	c1sccc1CCC(=O)Nc([nH]2)nnc2-c3ccc(cc3)OC	-2.4	5.2
Z296897106	c1ccc(F)cc1CC(=O)Nc([nH]2)nnc2-c3ccco3	14.2	19
Z1343274804	c1ccnc(c1C#N)NCCNc(nc2)ccc2-c(n3)[nH]c(=O)cc3C	2.7	5.3
Z1272615617	CC(C)(C)C(=O)NCCNc(nc1)ccc1-c(n2)[nH]c(=O)cc2CC	-3.4	4.6

Measurement of IC_{50}

Among the seven potential hit compounds, IC_{50} of Z820655914, Z1157725083, and Z57745307 were tried to be determined. The dose-response curves (DRC) were built using serial eight 2-fold dilution points in the range starting from 100 uM, where four wells were used. The DRCs shown in Figure S2 indicate that these compounds showed no inhibition in the range.

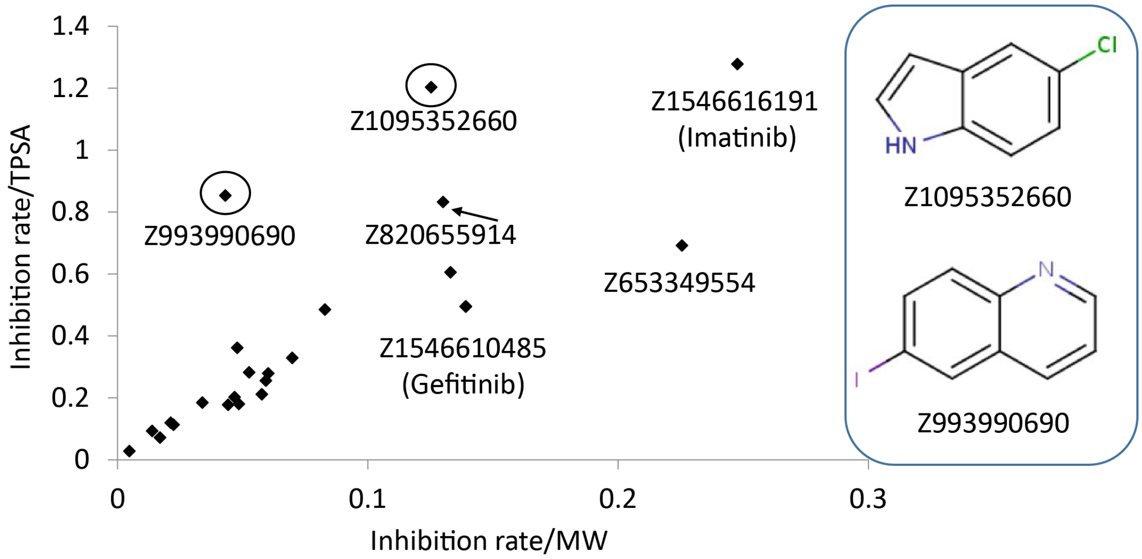

Supplementary Figure S1: Ligand efficiency indices: inhibition rate (%) divided by MW or topological polar surface area (TPSA)

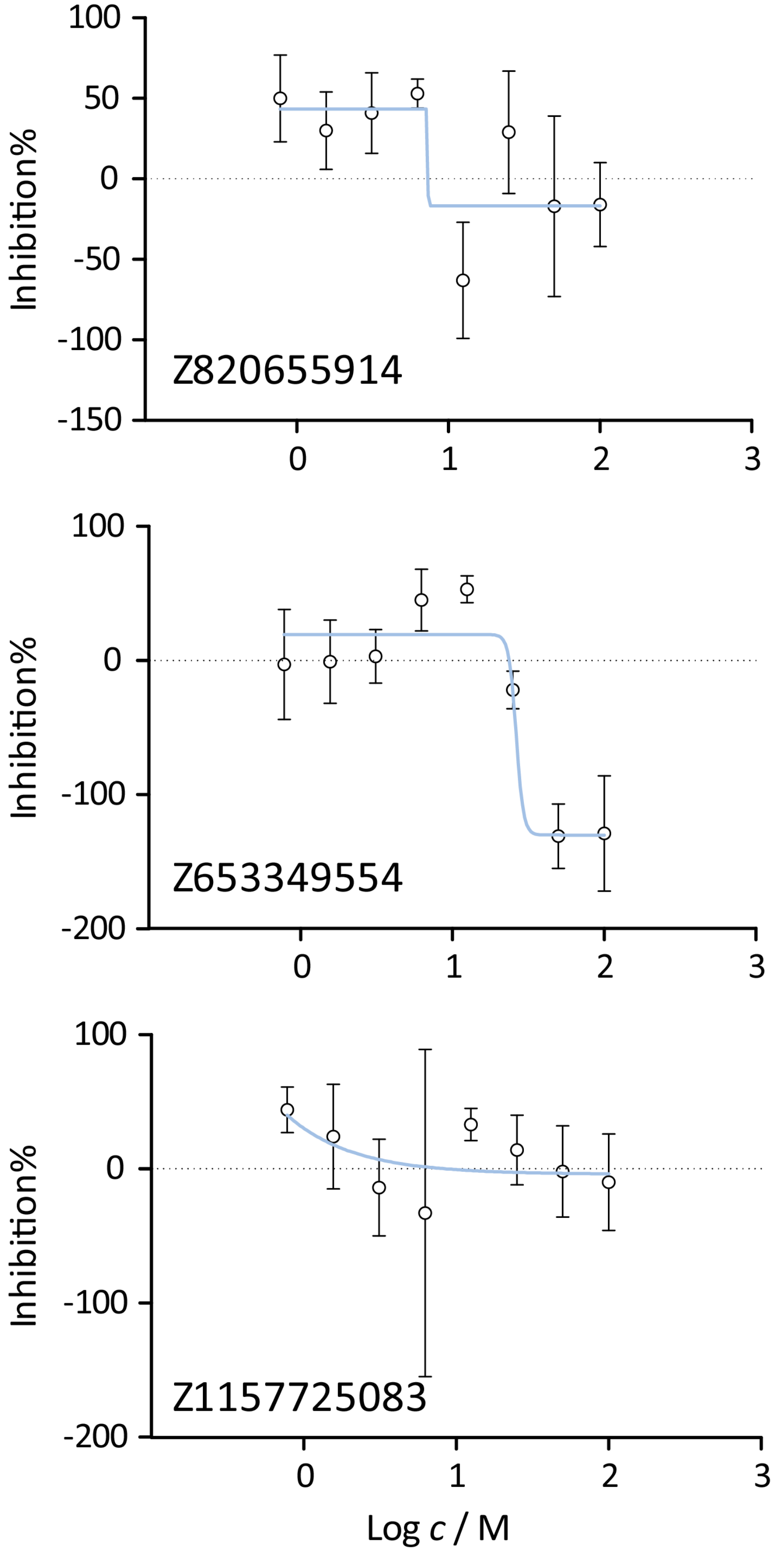

Supplementary Figure S2: Dose-response curves (DRC)
